# Supplementary material for: Rare de novo damaging DNA variants are enriched in attention-deficit/hyperactivity disorder and implicate risk genes
Source: Nat Commun. 2024 Jul 12;15:5870. doi: 10.1038/s41467-024-50247-7 (PMC11245598; doi:10.1038/s41467-024-50247-7)
Supplement: Supplementary file 1 — Supplementary Information [file 41467_2024_50247_MOESM1_ESM.pdf]

Supplementary Information for "Rare *de novo* damaging DNA variants are enriched in attention-deficit/hyperactivity disorder and implicate risk genes"

File Includes:

- Supplementary Table 1
- Supplementary Figures 1-3
- Supplementary Methods
- Supplementary Discussion
- Supplementary References

Supplementary Table 1. Distribution of classes of rare and ultra-rare *de novo* variants in ADHD cases and controls

	**Variant counts**		**Mutation rate (x10-8) per basepair (95% CI)[b]**		**Estimated coding variants per individual (95% CI)[c]**		**Rate Ratio (95% CI)[d]**	***p*-value**
	**ADHD case (n=147)**	**Control (n=780)**	**ADHD case (n=147)**	**Control (n=780)**	**ADHD case (n=147)**	**Control (n=780)**		
**Rare *de novo* variant class (non-neuro gnomAD AF<0.001)[a]**								
Synonymous SNVs	32	166	0.35 (0.24-0.50)	0.45 (0.38-0.52)	0.23 (0.16-0.32)	0.29 (0.25-0.34)	0.79 (0.56-1.10)	0.91
All missense (Mis)[e]	95	430	1.05 (0.85-1.28)	1.16 (1.05-1.27)	0.68 (0.55-0.83)	0.75 (0.68-0.83)	0.91 (0.75-1.10)	0.82
Missense with MPC 0-1 (Mis-B)[f]	68	308	0.75 (0.58-0.95)	0.83 (0.74-0.93)	0.49 (0.38-0.62)	0.54 (0.48-0.60)	0.91 (0.72-1.13)	0.79
Missense with MPC 1-2 (Mis-P)[g]	15	76	0.17 (0.09-0.27)	0.20 (0.16-0.26)	0.11 (0.06-0.18)	0.13 (0.10-0.17)	0.81 (0.48-1.31)	0.81
Missense with MPC >2 (Mis-D)[h]	7	17	0.08 (0.03-0.16)	0.05 (0.03-0.07)	0.05 (0.02-0.10)	0.03 (0.02-0.05)	1.69 (0.70-3.77)	0.18
All PTV[i]	17	42	0.19 (0.10-0.30)	0.13 (0.08-0.15)	0.12 (0.07-0.17)	0.07 (0.05-0.10)	1.66 (0.98-2.74)	0.06
PTV frameshift indels	7	23	0.08 (0.03-0.16)	0.06 (0.04-0.09)	0.05 (0.02-0.10)	0.04 (0.03-0.06)	1.25 (0.53-2.67)	0.37
PTV stopgain	9	19	0.10 (0.05-0.19)	0.05 (0.03-0.08)	0.06 (0.03-0.12)	0.03 (0.02-0.05)	1.94 (0.89-4.00)	0.08
PTV splicing	1	0	0.01 (0.00-0.06)	0.00 (0.00-0.01)	0.01 (0.00-0.04)	0.00 (0.00-0.01)	Inf (0.22-Inf)	0.20
Nonframeshift indels	3	7	0.03 (0.01-0.10)	0.02 (0.01-0.04)	0.02 (0.00-0.06)	0.01 (0.00-0.03)	1.76 (0.39-6.33)	0.31
Damaging (PTV + Mis-D)	24	59	0.27 (0.17-0.40)	0.16 (0.12-0.21)	0.17 (0.11-0.26)	0.10 (0.08-0.13)	1.67 (1.08-2.53)	**0.03**
All[j]	148	652	1.64 (1.38-1.92)	1.76 (1.63-1.90)	1.06 (0.90-1.25)	1.14 (1.06-1.23)	0.93 (0.80-1.08)	0.80
**Ultra-rare *de novo* variant class (non-neuro gnomAD AF<0.00005)[a]**								
synonymous SNVs	21	113	0.23 (0.14-0.36)	0.30 (0.25-0.37)	0.15 (0.09-0.23)	0.20 (0.16-0.24)	0.76 (0.49-1.14)	0.90
All missense (Mis)[k]	73	344	0.81 (0.63-1.02)	0.93 (0.83-1.03)	0.52 (0.41-0.66)	0.60 (0.54-0.67)	0.87 (0.70-1.08)	0.87
Missense with MPC 0-1 (Mis-B)[f]	50	243	0.55 (0.41-0.73)	0.66 (0.58-0.74)	0.36 (0.27-0.47)	0.43 (0.37-0.48)	0.84 (0.64-1.10)	0.88
Missense with MPC 1-2 (Mis-P)[g]	13	62	0.14 (0.08-0.25)	0.17 (0.13-0.21)	0.09 (0.05-0.16)	0.11 (0.08-0.14)	0.86 (0.48-1.45)	0.73
Missense with MPC >2 (Mis-D)[h]	7	15	0.08 (0.03-0.16)	0.04 (0.02-0.07)	0.05 (0.02-0.1)	0.03 (0.01-0.04)	1.91 (0.78-4.36)	0.12
All PTV[i]	17	36	0.19 (0.11-0.30)	0.10 (0.07-0.13)	0.12 (0.07-0.2)	0.06 (0.04-0.09)	1.94 (1.13-3.24)	**0.02**
PTV frameshift indels	7	21	0.08 (0.03-0.16)	0.06 (0.04-0.09)	0.05 (0.02-0.10)	0.04 (0.02-0.06)	1.37 (0.6-2.96)	0.30
PTV stopgain	9	15	0.1 (0.05-0.19)	0.04 (0.02-0.07)	0.06 (0.03-0.12)	0.03 (0.01-0.04)	2.46 (1.1-5.28)	0.03
PTV splicing	1	0	0.01 (0.00-0.06)	0.00 (0.00-0.01)	0.01 (0.00-0.04)	0.00 (0.00-0.01)	Inf (0.22-Inf)	0.20
Nonframeshift indels	1	5	0.01 (0.00-0.06)	0.01 (0.00-0.03)	0.01 (0.00-0.04)	0.01 (0.00-0.02)	0.82 (0.04-5.71)	0.73
Damaging (PTV+Mis-D)	24	51	0.27 (0.17-0.40)	0.14 (0.10-0.18)	0.17 (0.11-0.26)	0.09 (0.07-0.12)	1.93 (1.24-2.97)	**0.007**
All[l]	113	504	1.25 (1.03-1.50)	1.36 (1.24-1.48)	0.81 (0.67-0.98)	0.88 (0.81-0.96)	0.92 (0.77-1.10)	0.80

gnomAD, the Genome Aggregation Database; AF, allele frequency; CI, confidence interval; SNVs, single nucleotide variants; PTV, protein-truncating variants; indel, insertion-deletion variants; Mis-B, benign missense variants; Mis-P, possibly damaging missense variants; Mis-D, damaging missense variants.

[a]Variants were annotated with ANNOVAR using RefSeq hg19 definitions.

[b]*De novo* mutation rates were calculated as the number of variants divided by the number of haploid “callable” base pairs.

[c]The estimated number of *de novo* mutations per individual was calculated by multiplying by the size of the RefSeq hg19 coding exome (32,471,596 bp).

[d]Rates were compared using a one-sided rate ratio test with p-value <0.05 considered significant. Bold indicates significant p-values.70

[e]All rare missense variants contain five rare missense variants in ADHD cases and 29 rare in control trios that were not annotated by the "missense badness, PolyPhen-2, constraint" scores (MPC).
[f]Missense variants with an MPC score of 0-1.
[g]Missense variants with an MPC score of 1-2.
[h]Missense variants with an MPC score of >2.
[i]Includes frameshift indels, premature stop codons, and canonical splice site variants
[j]All rare annotated coding variants, which include one variant annotated as unknown in ADHD cases and 7 in controls.
[k]All ultra-rare missense variants contain three missense variants in ADHD cases and 24 in control trios that were not annotated by the "missense badness, PolyPhen-2, constraint" scores (MPC).
[l]All ultra-rare annotated coding variants, which includes one variant annotated as unknown in ADHD cases and 6 in controls.

Supplementary Figure 1. Plots from principal component analysis to identify outliers based on whole-exome DNA sequencing metrics

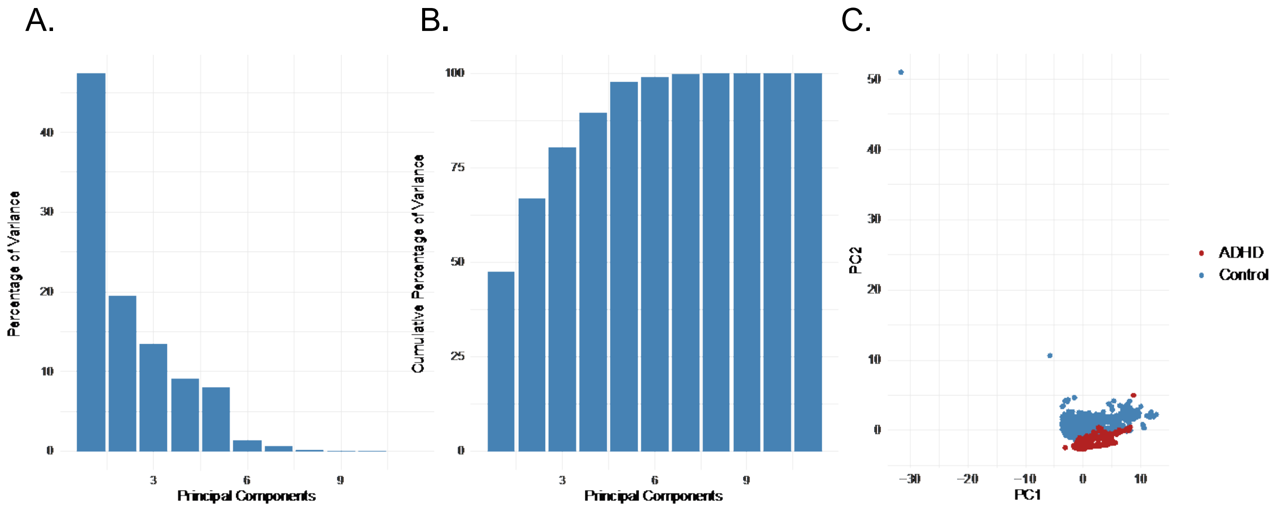

Supplementary Figure 1. Plots from the principal components analysis (PCA). (A) Shows the percentage of variance captured by the 11 principal components from the exome metrics data from cases and controls. (B) Shows the cumulative percentage of variance captured by these components and demonstrates that over 75% of the cumulative variance was captured by the first three principal components. (C) Shows the first two principal components based on the PCA of the exome sequencing quality metrics. ADHD cases are plotted in red, and controls in blue. This figure includes PCA outliers (>5 standard deviations in PCAs 1-3) removed during the quality control.

Supplementary Figure 2. Visualization of aligned sequencing reads for putative rare and ultra-rare *de novo* damaging variants identified in ADHD parent-child trios. These 24 damaging *de novo* variants passed *in silico* confirmation and were included in burden and downstream analyses.

A. Chr1:45251759CAGAG>C (frameshift deletion) for ADHD44.p1, ADHD44.fa, and ADHD44.mo.

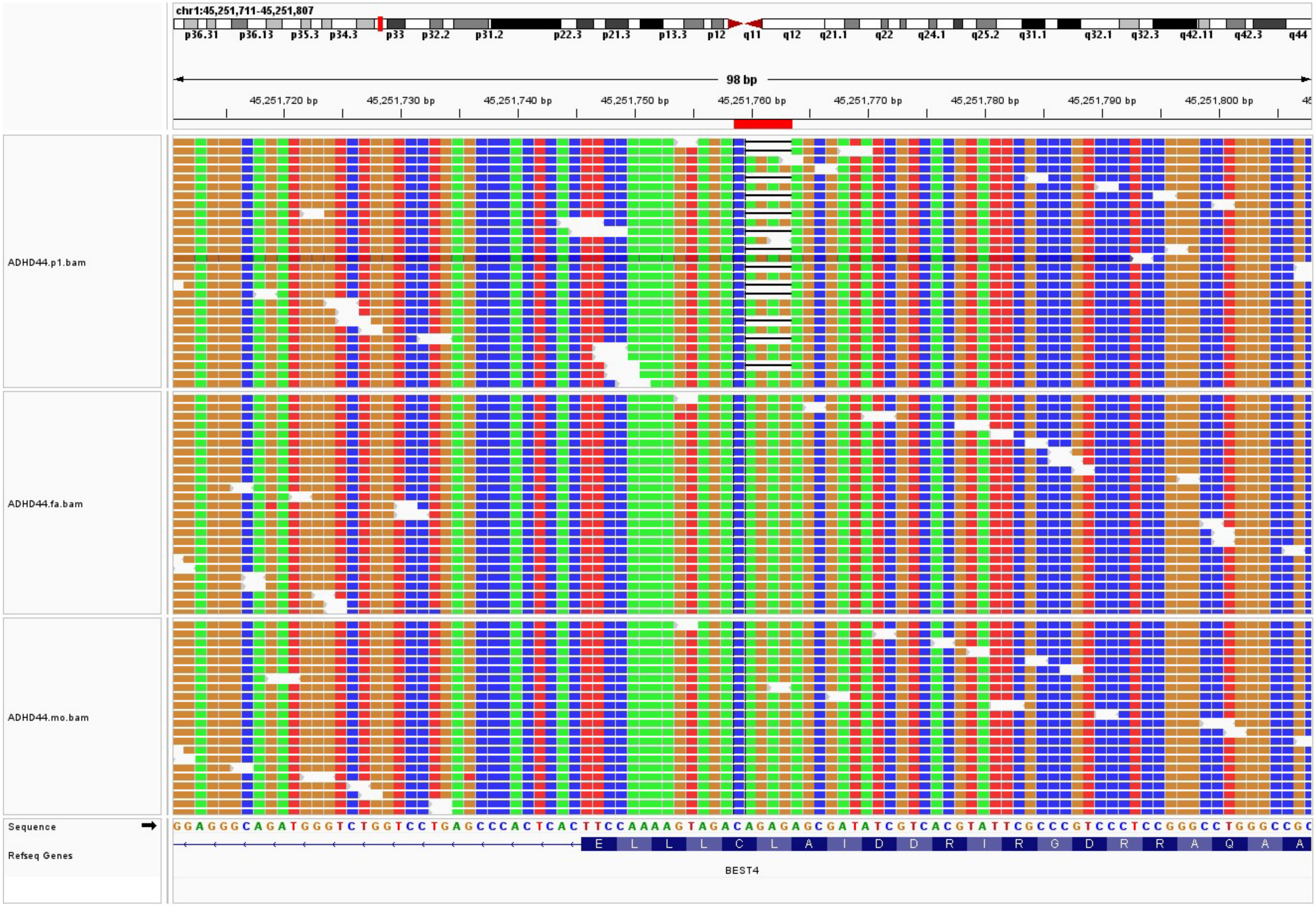

B. Chr11:65623472T>C (missense) for ADHD69.p1, ADHD69.fa, and ADHD69.mo.

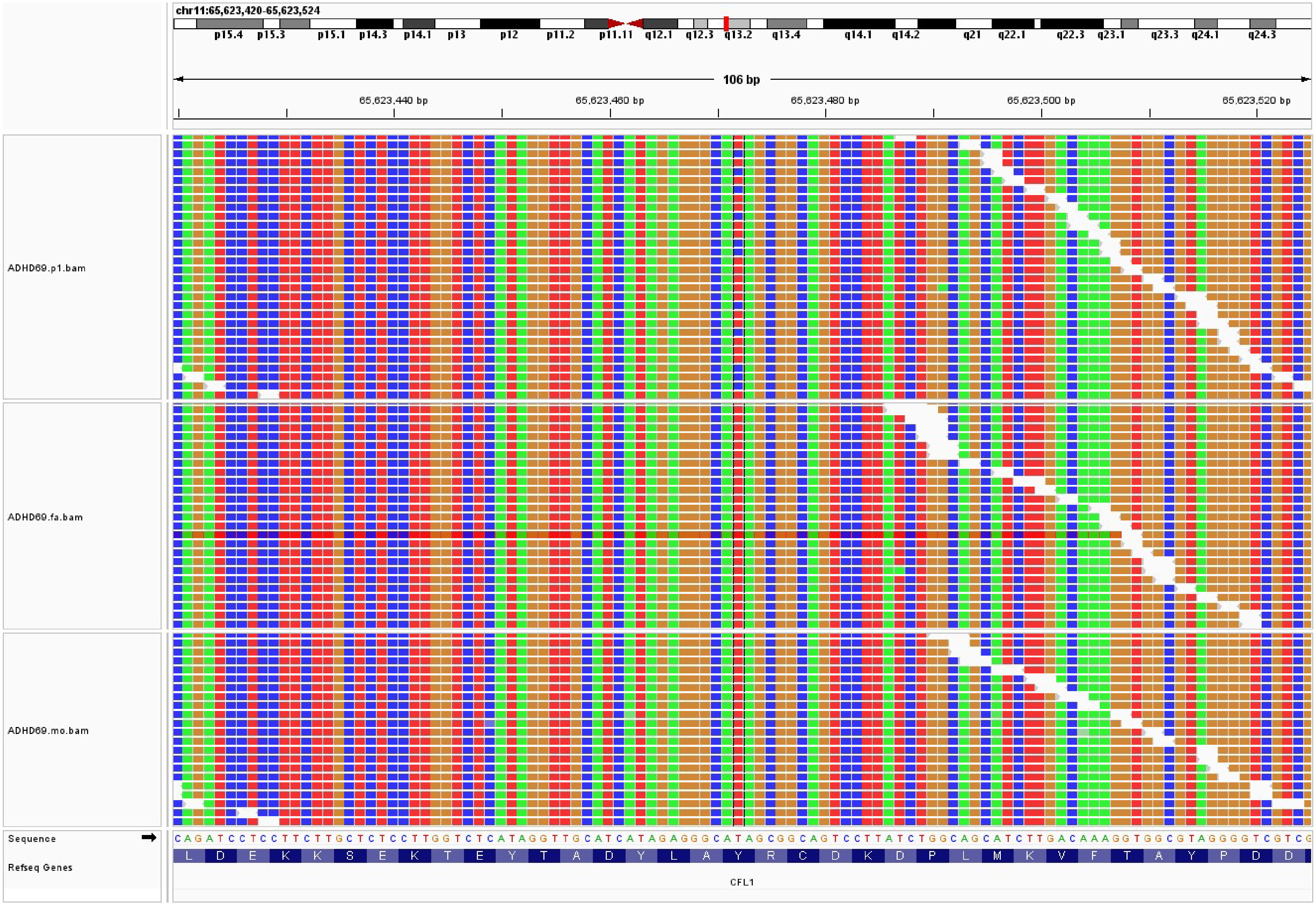

C. Chr10:125804342G>GGT (frameshift insertion) for ADHD95.p1, ADHD95.fa, ADHD95.mo.

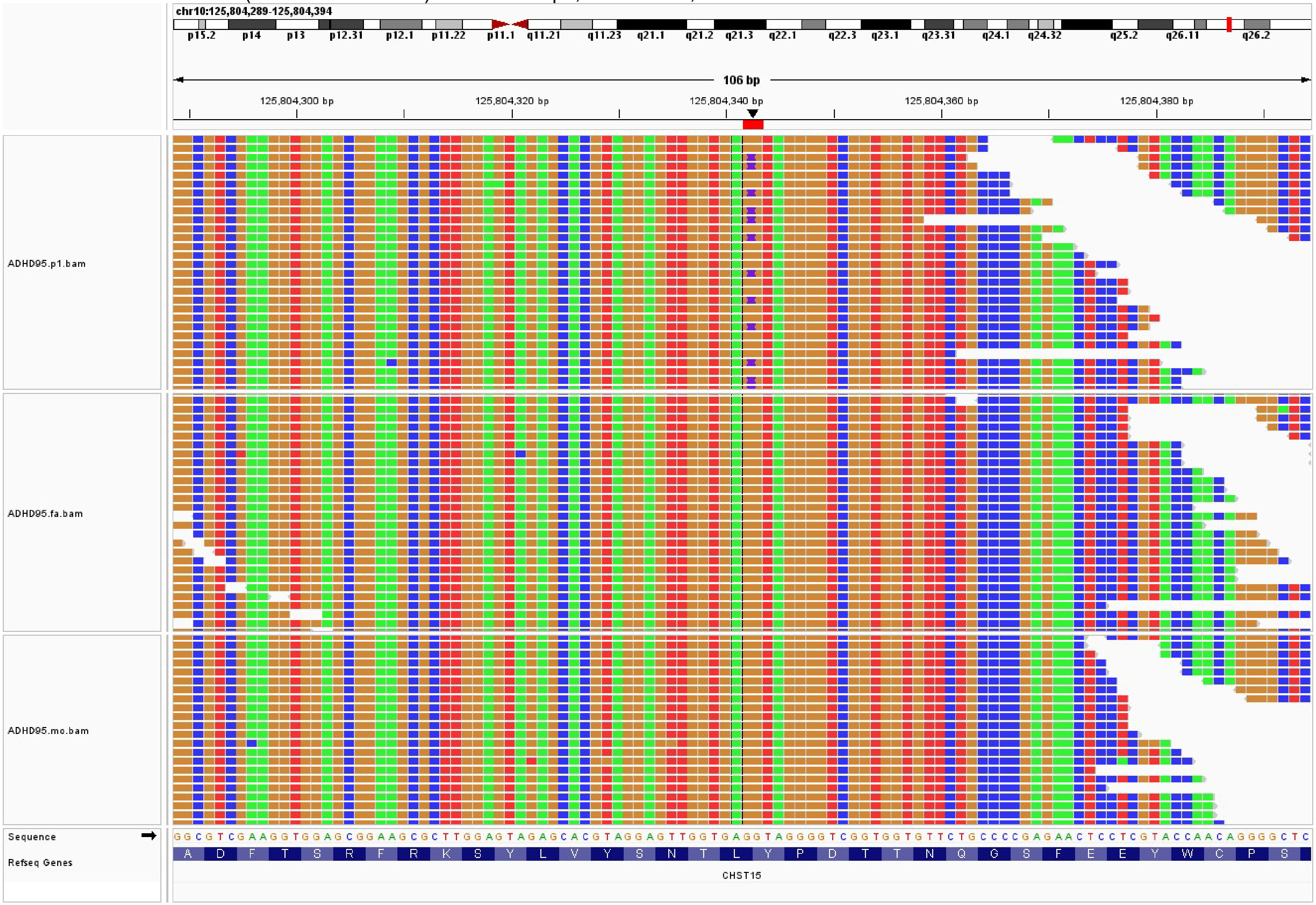

D. Chr2:80808942C>T (missense) for ADHD37.p1, ADHD37.fa, and ADHD37.mo.

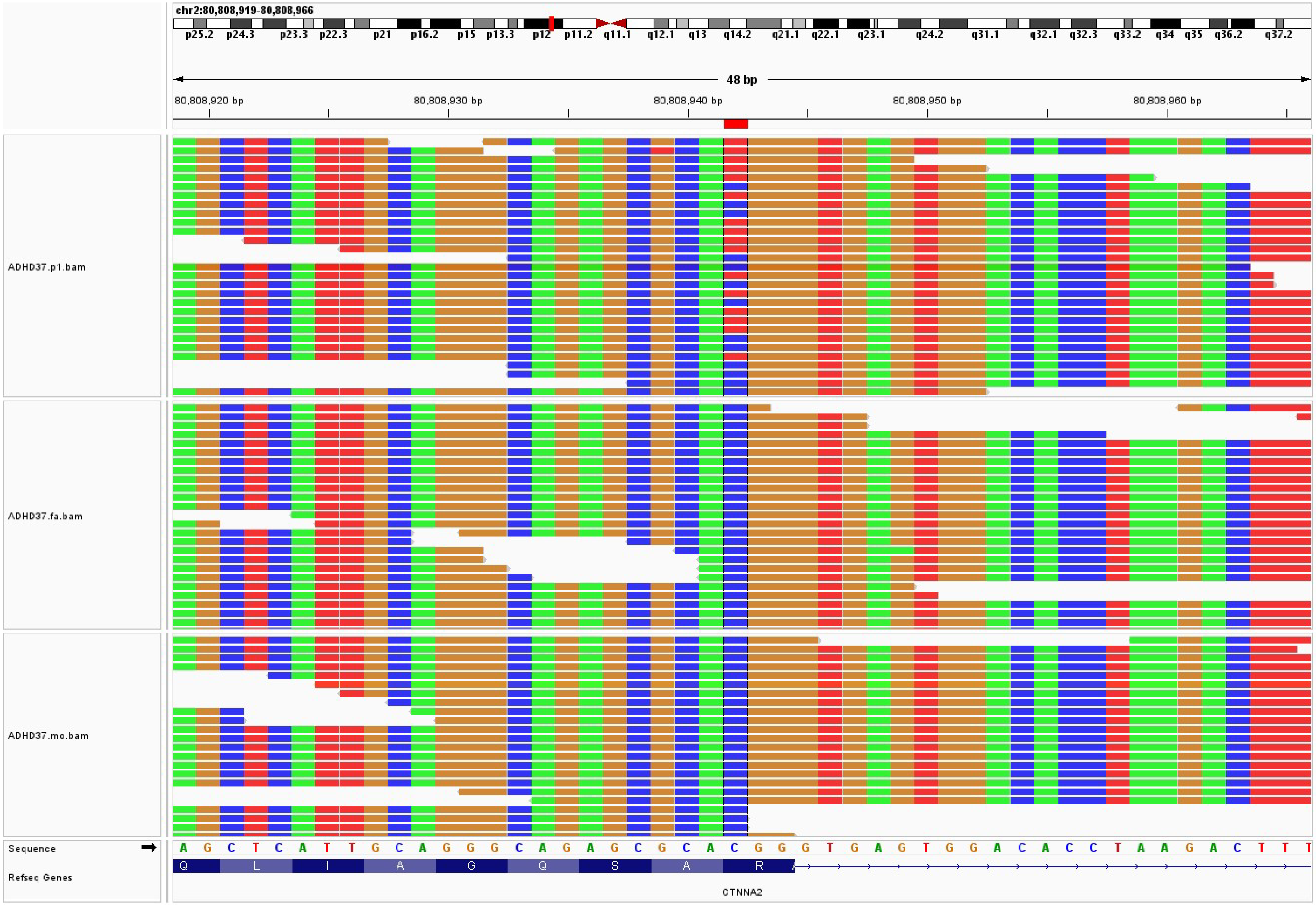

E. Chr5:11236867AC>A (frameshift deletion) for ADHD107.p1, ADHD107.fa, and ADHD107.mo.

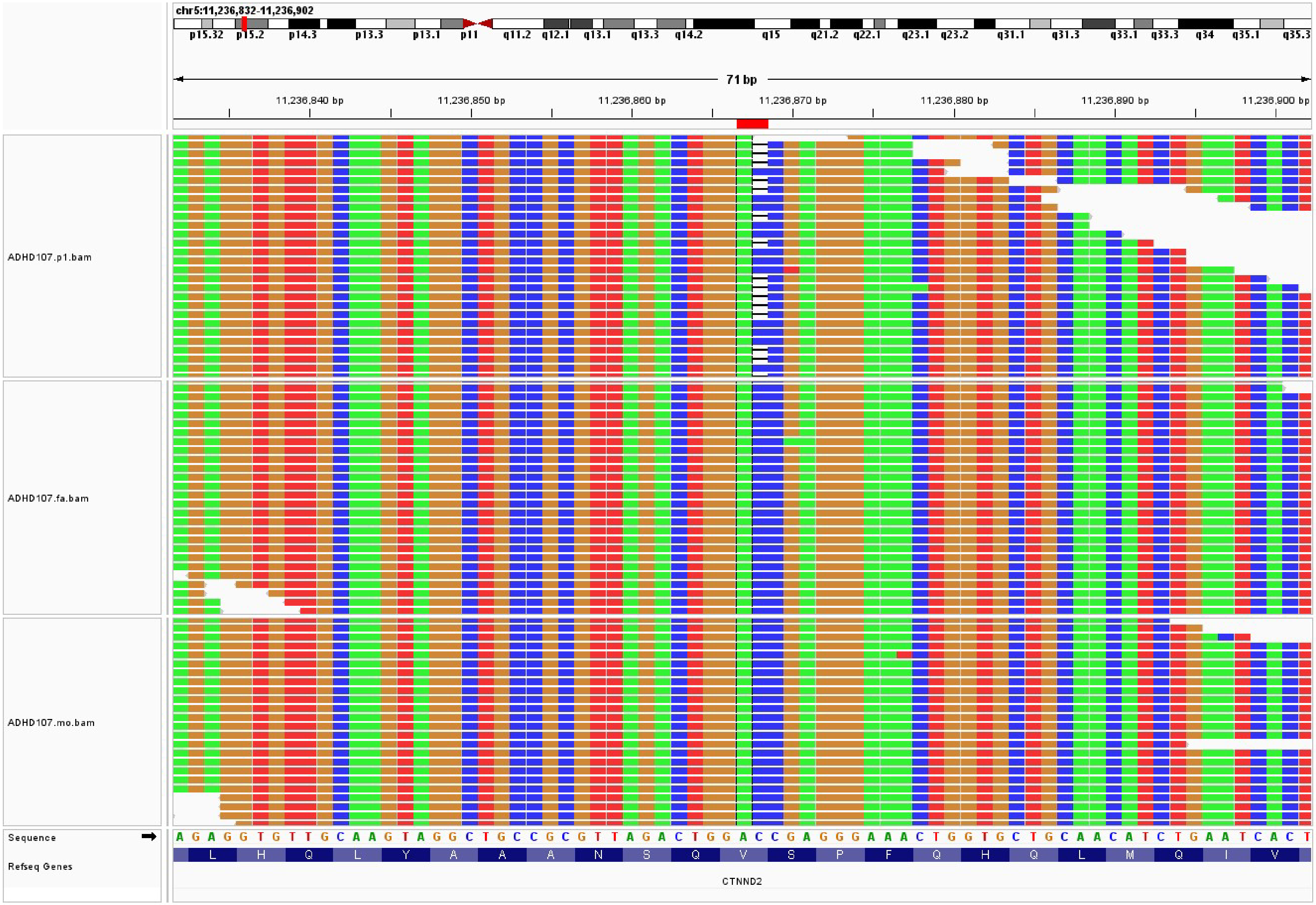

F. Chr11:65359271CATGG>C (frameshift deletion) for ADHD98.p1, ADHD98.fa, and ADHD98.mo.

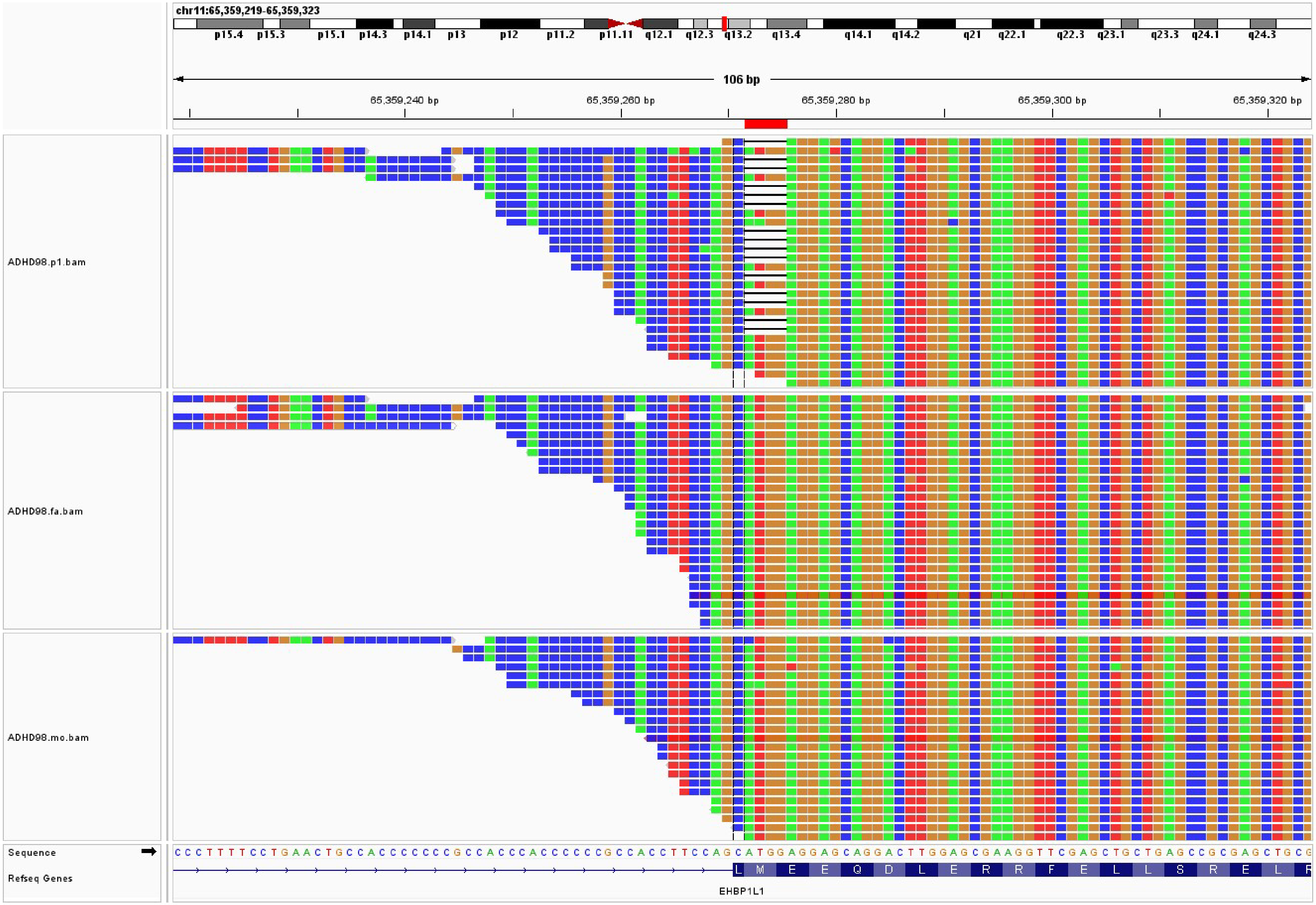

G. Chr2:55071273C>T (stopgain) for ADHD61.p1, ADHD61.fa, and ADHD61.mo.

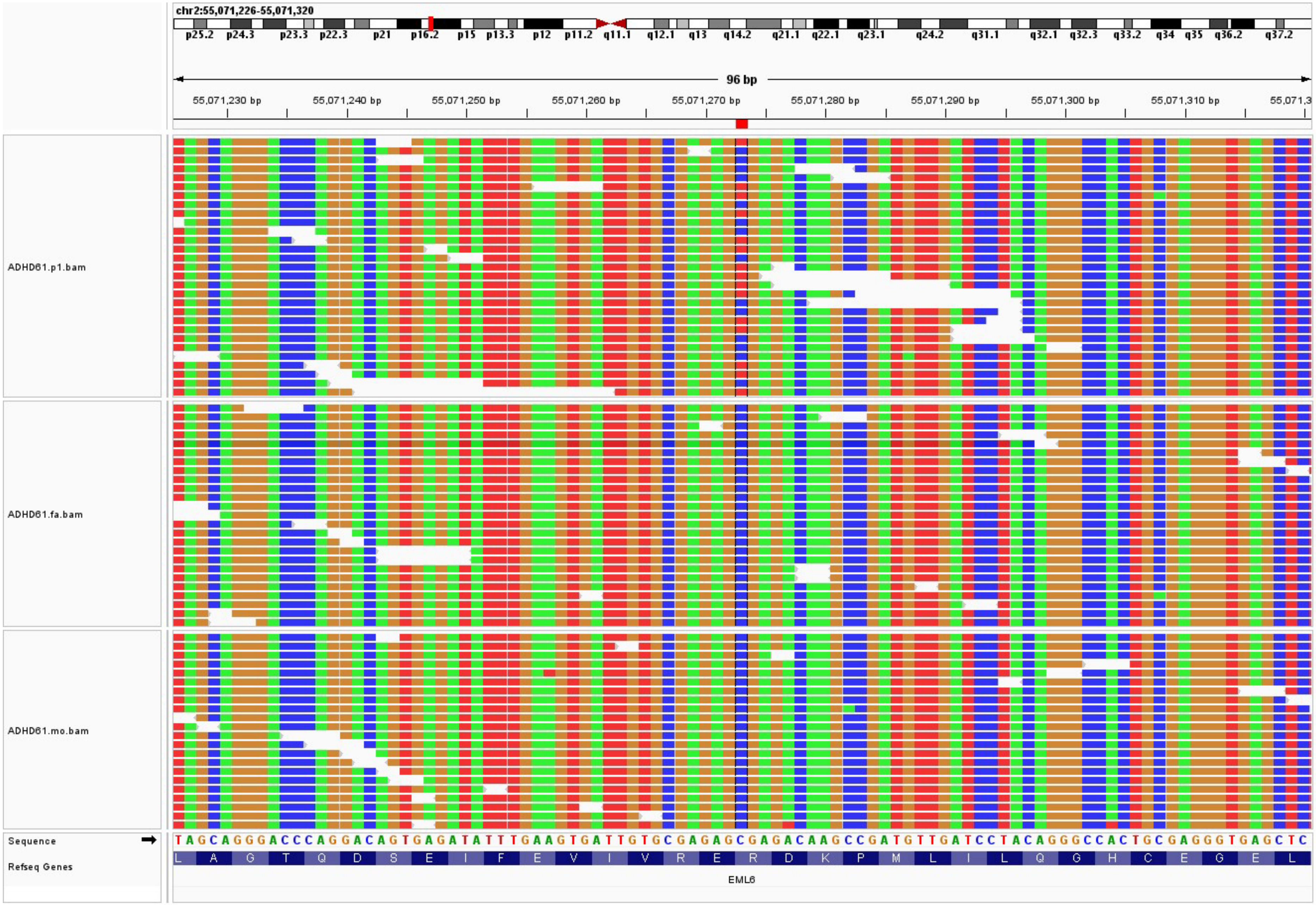

H. Chr2:48035289G>A (missense) for ADHD86.p1, ADHD86.fa, and ADHD86.mo.

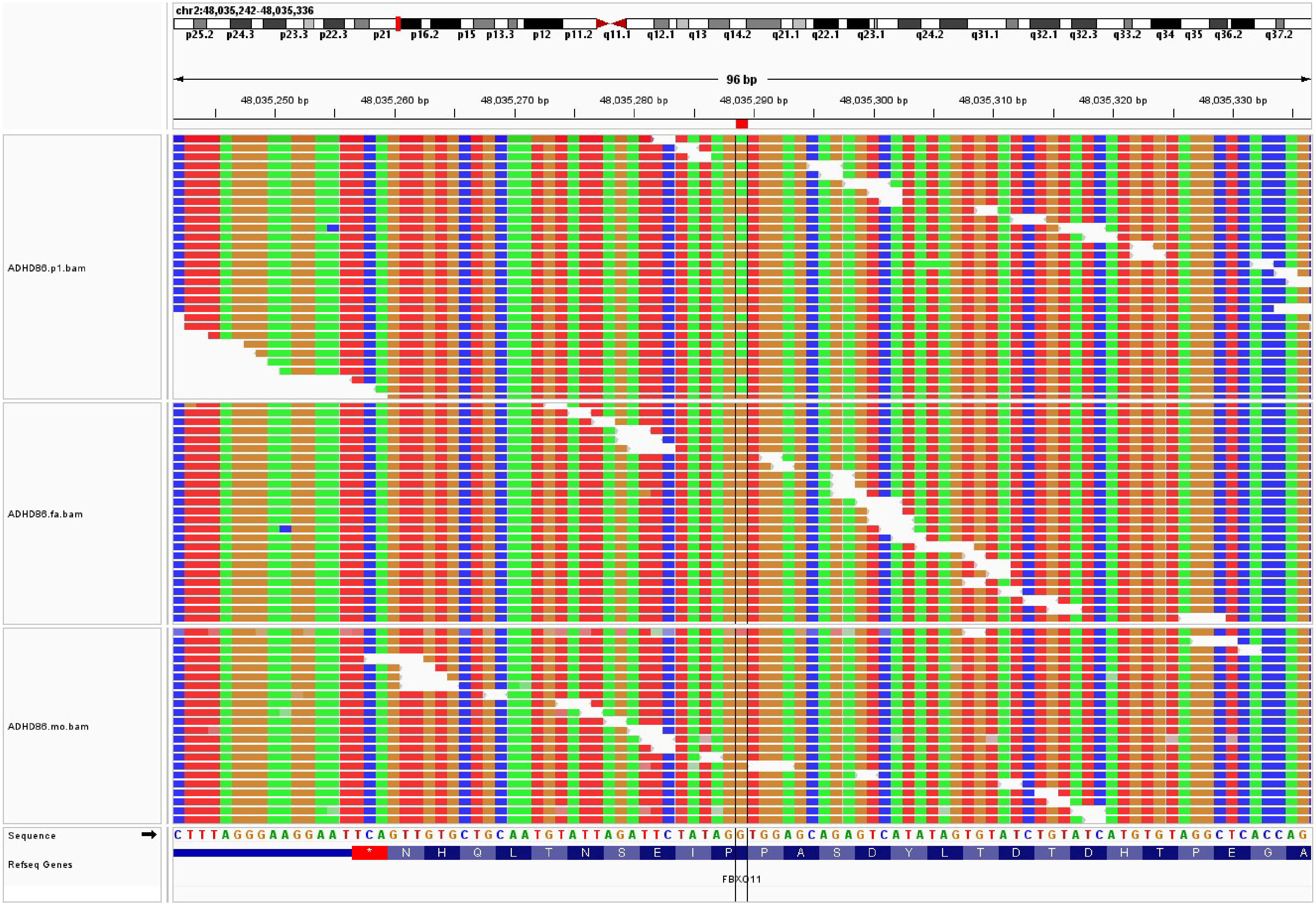

I. Chr5:180665146T>C (missense) for ADHD134.p1, ADHD134.fa, and ADHD134.mo.

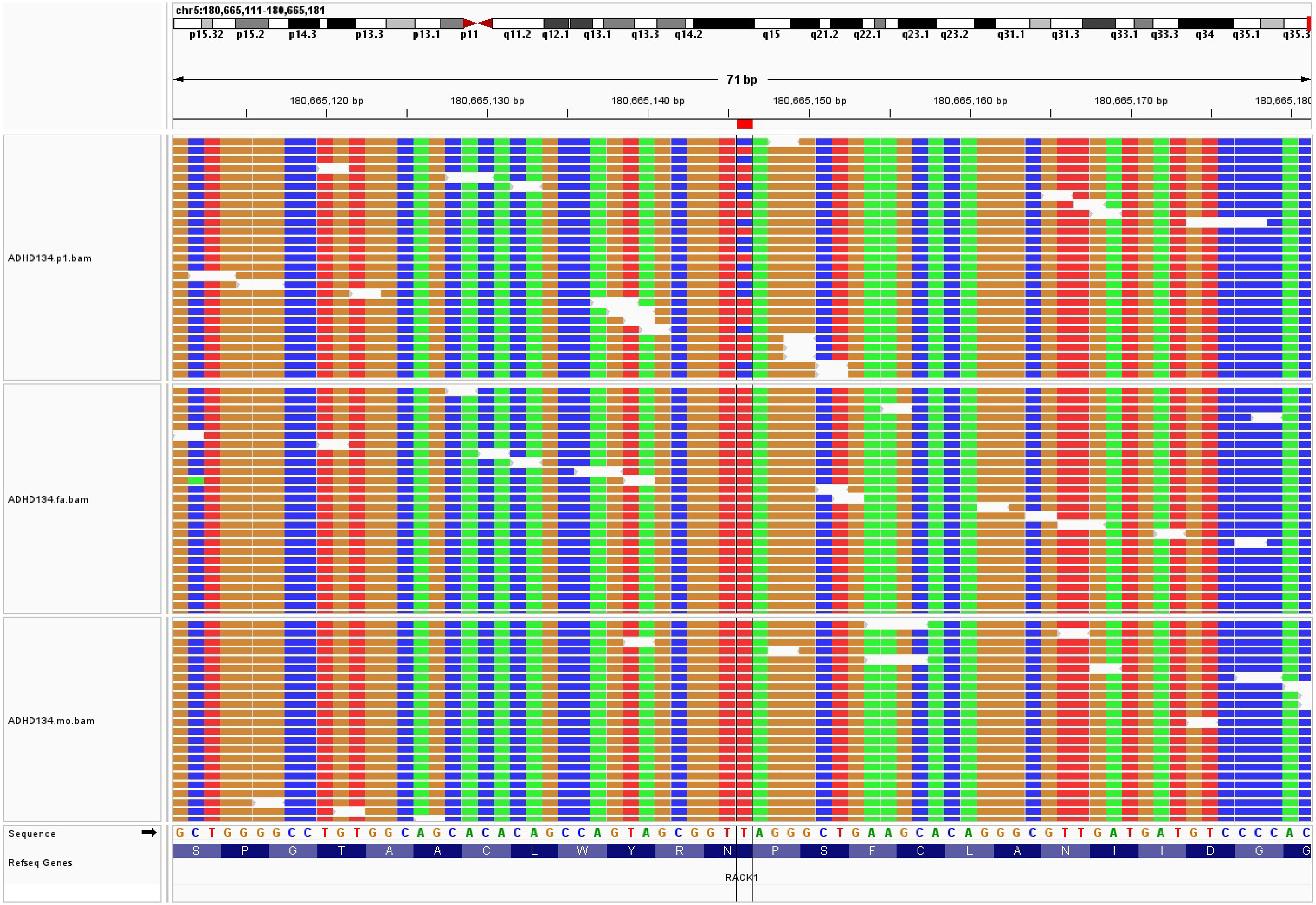

J. Chr3:121435768A>C (stopgain) for ADHD117.p1, ADHD117.fa, and ADHD117.mo.

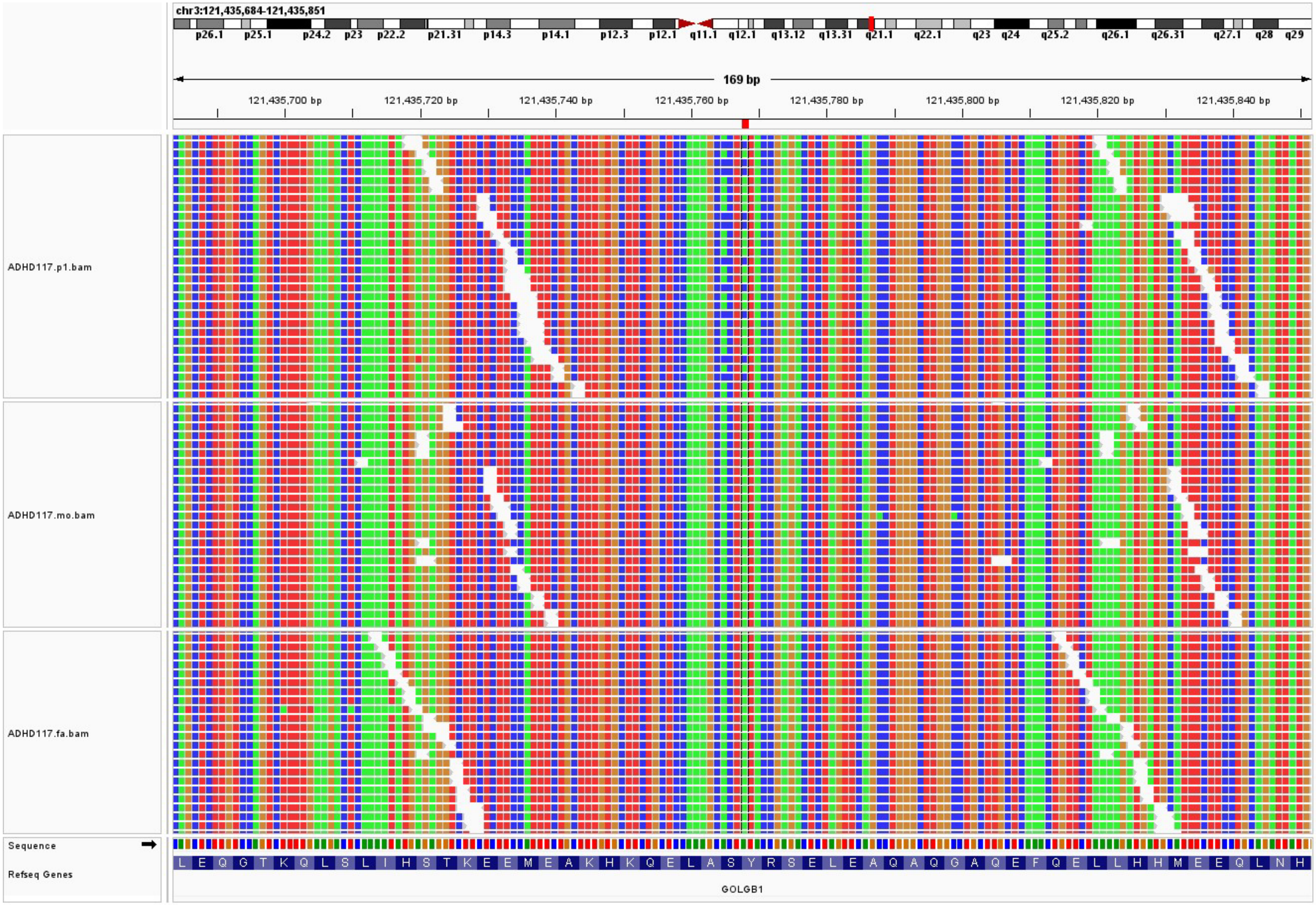

K. Chr1:202704703G>A (stopgain) for ADHD50.p1, ADHD50.fa, and ADHD50.mo.

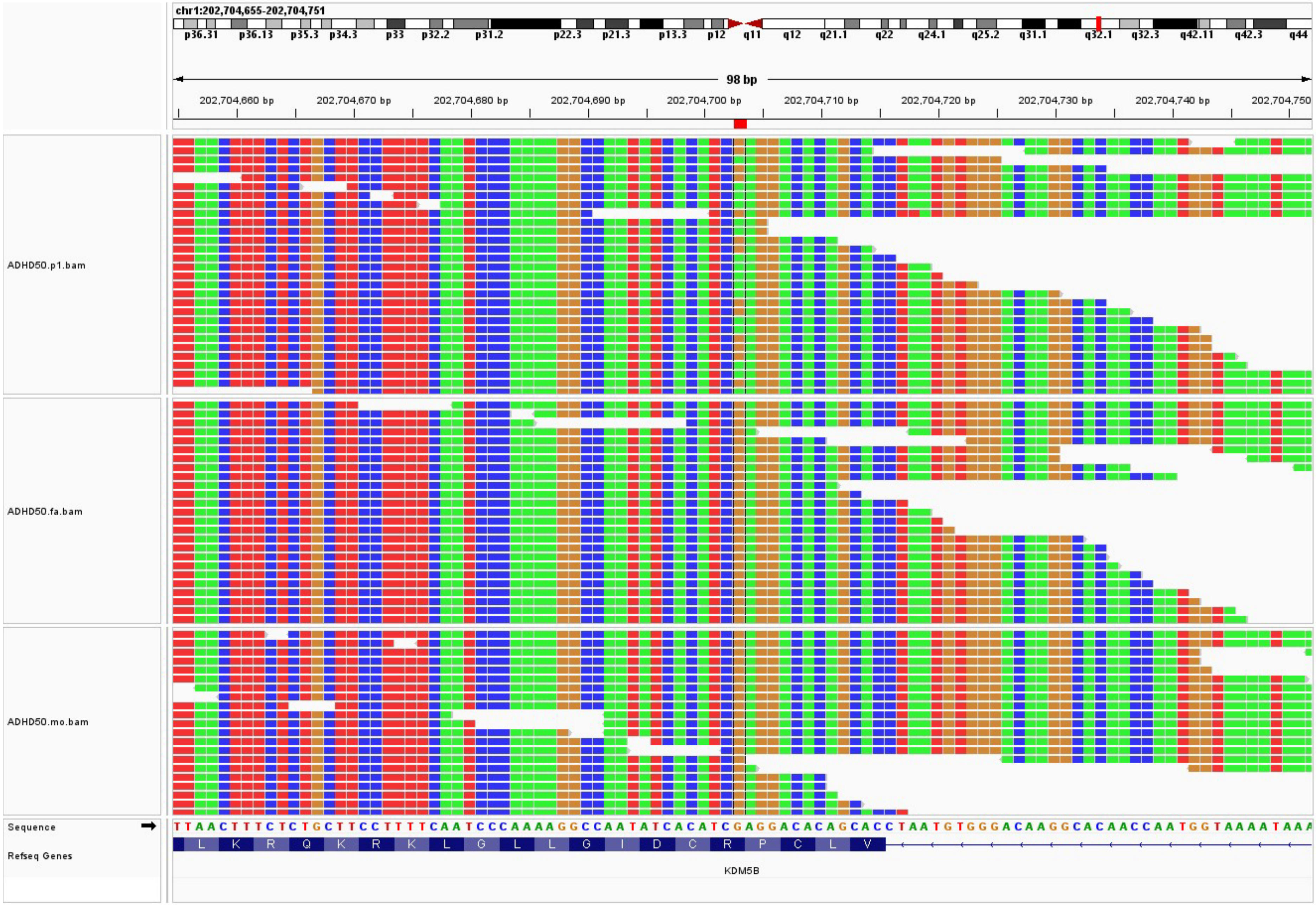

L. Chr1:202711840TC>T (frameshift deletion) for ADHD58.p1, ADHD58.fa, and ADHD58.mo.

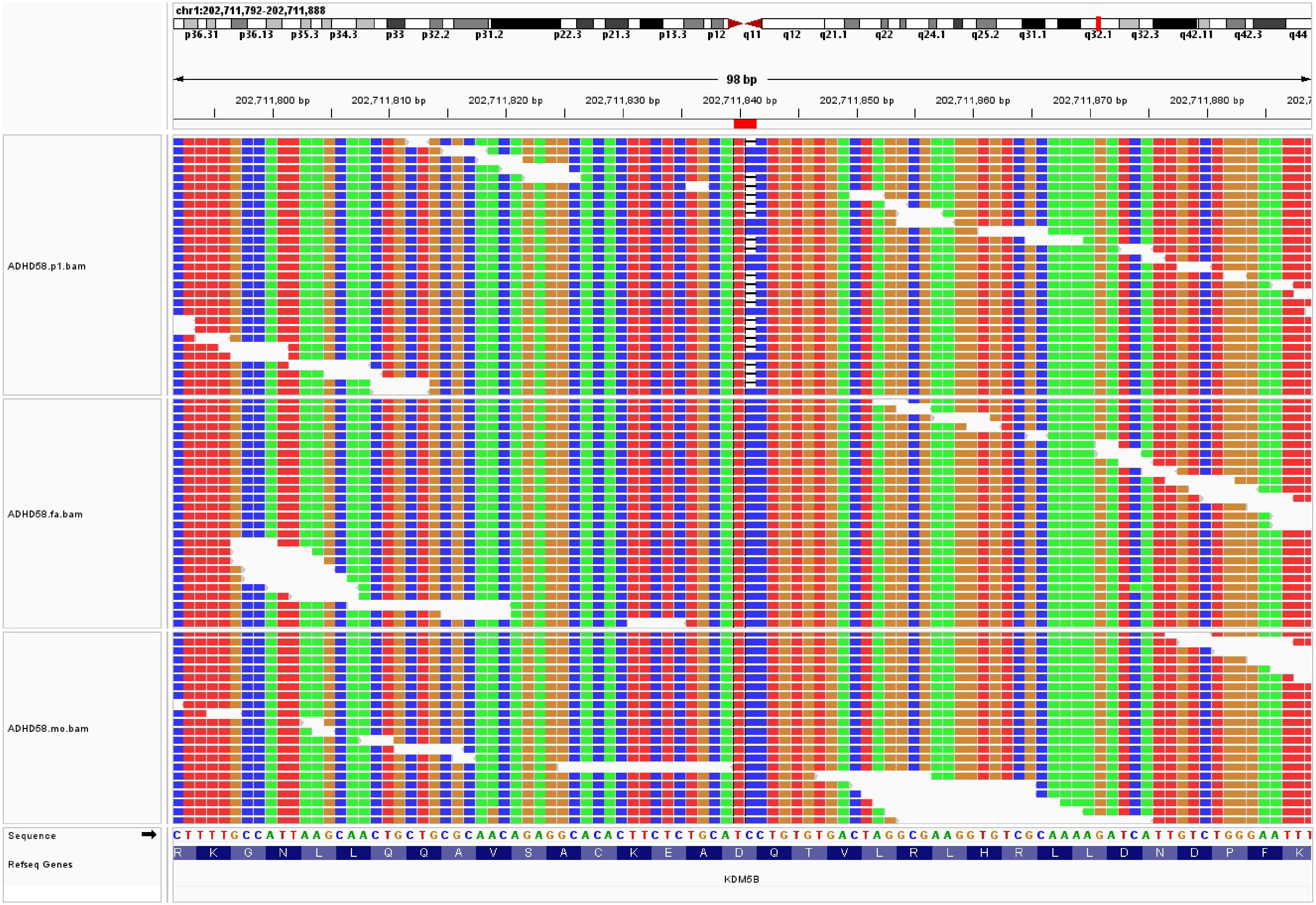

M. Chr1:62676185C>A (stopgain) for ADHD130.p1, ADHD130.fa, and ADHD.mo.

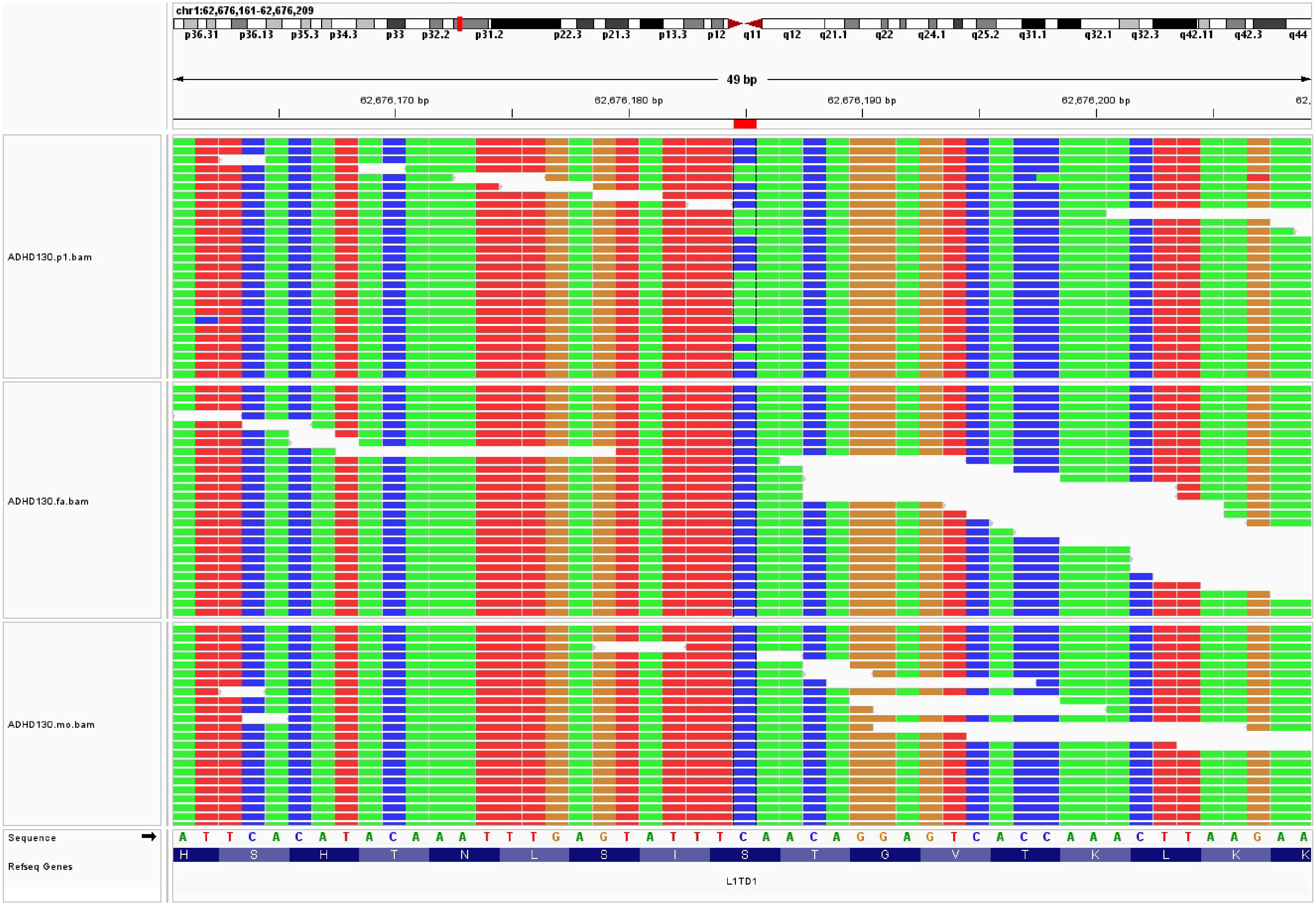

N. Chr11:78282471G>A (stopgain) for ADHD33.p1, ADHD33.fa, and ADHD33.mo.

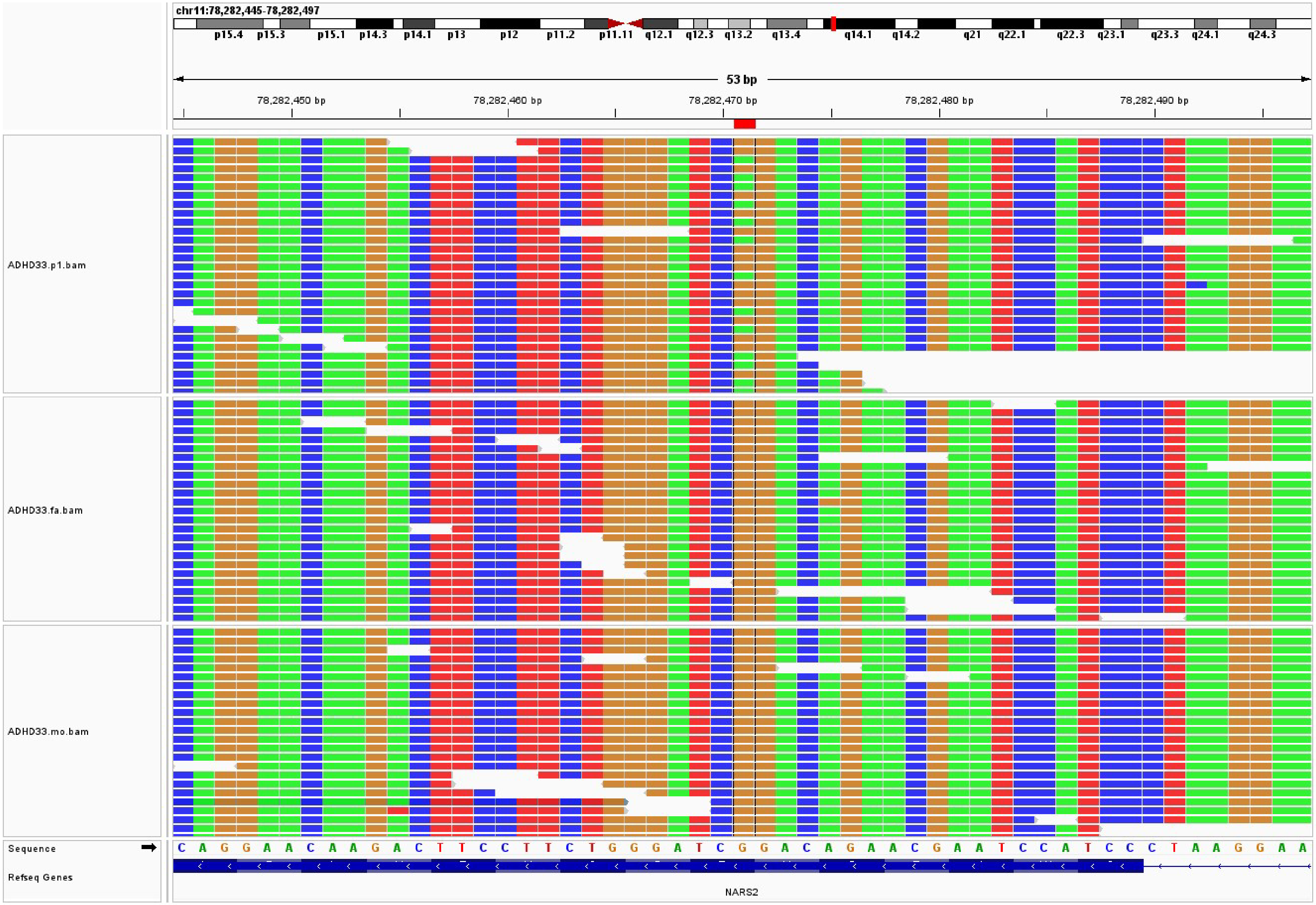

O. Chr19:17337532G>T (stopgain) for ADHD71.p1, ADHD71.fa, and ADHD71.mo.

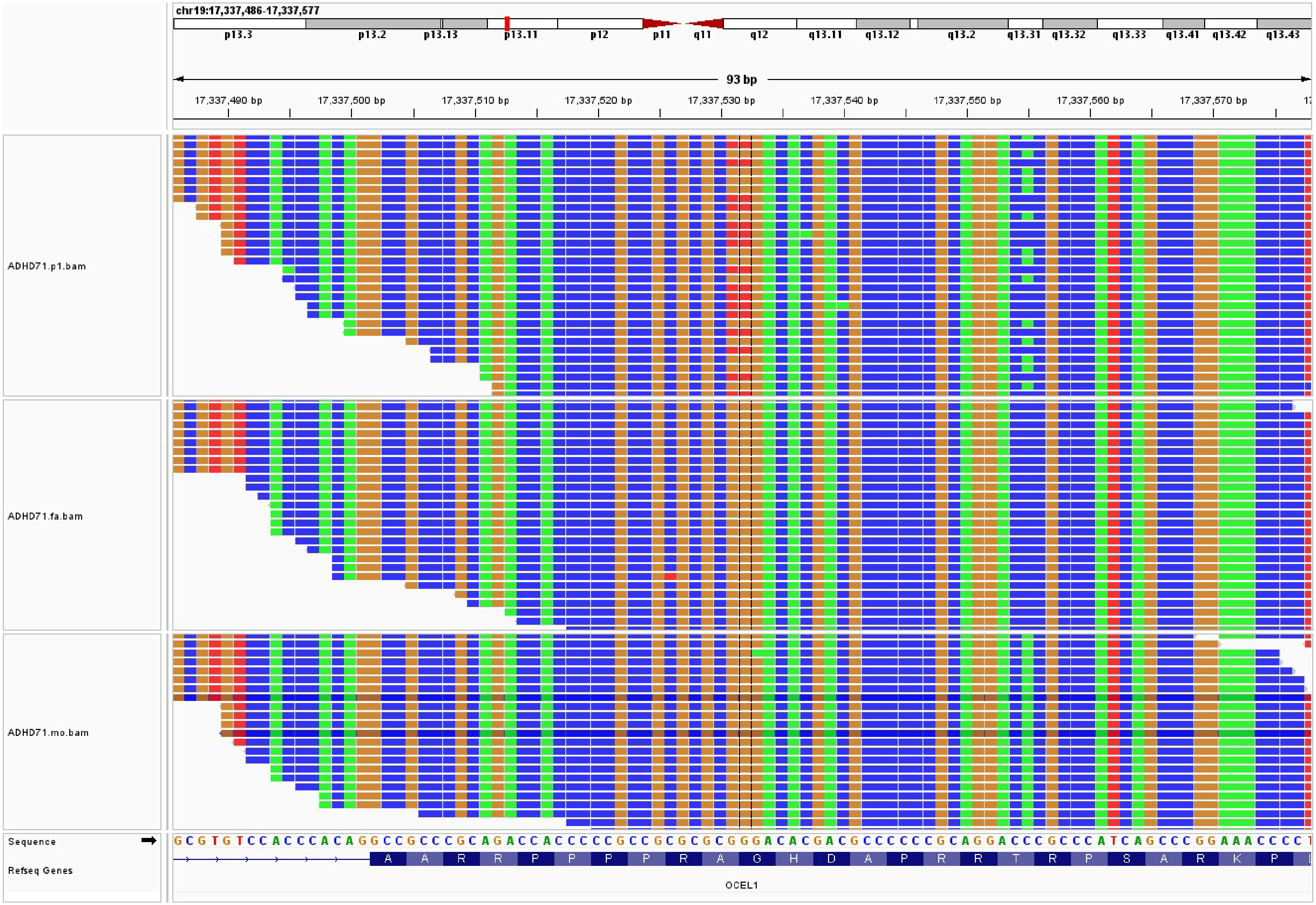

P. Chr11:77103522G>A (missense) for ADHD141.p1, ADHD141.fa, and ADHD141.mo.

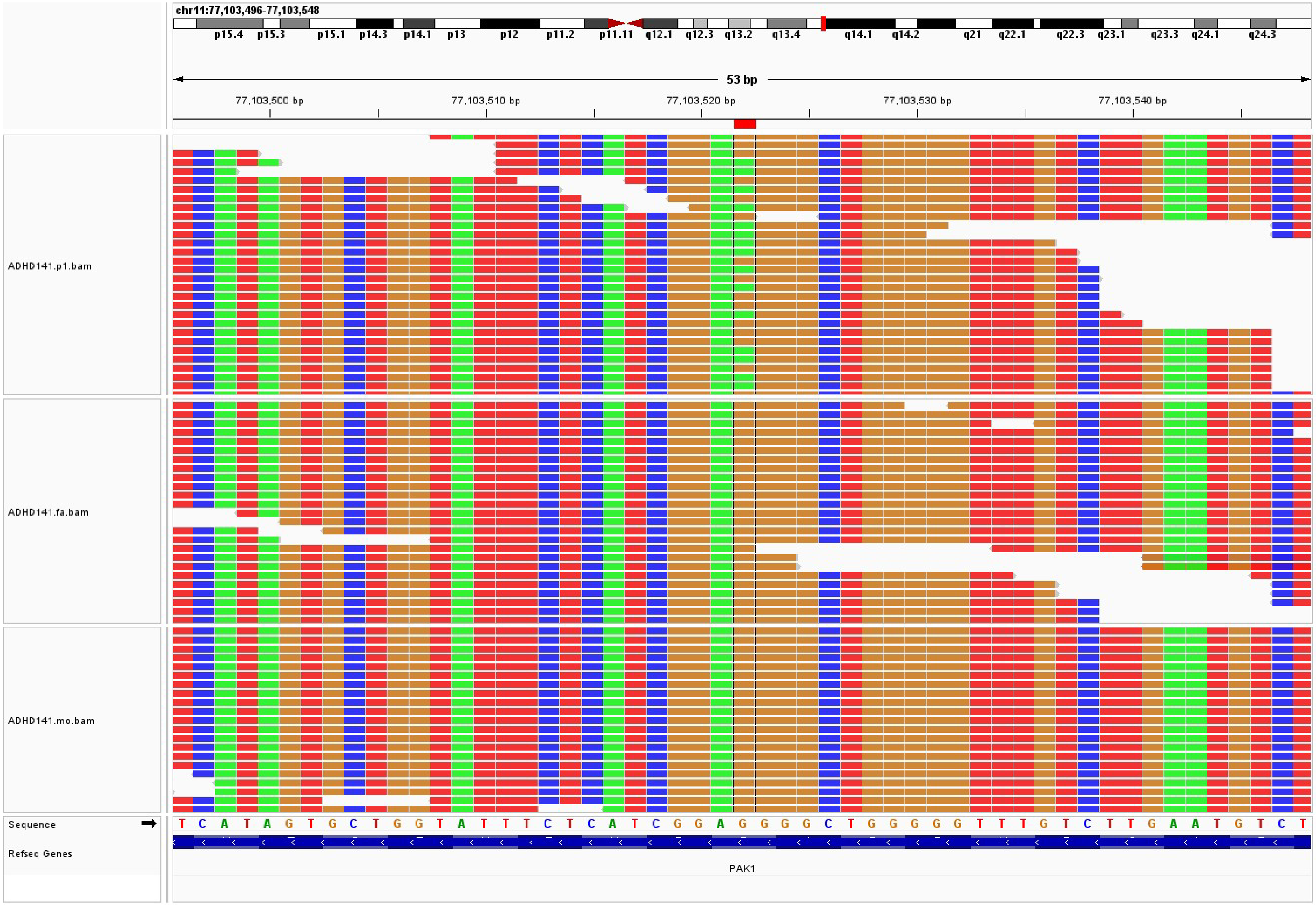

Q. Chr1:242511463TG>T (frameshift deletion) for ADHD84.p1, ADHD84.fa, and ADHD84.mo.

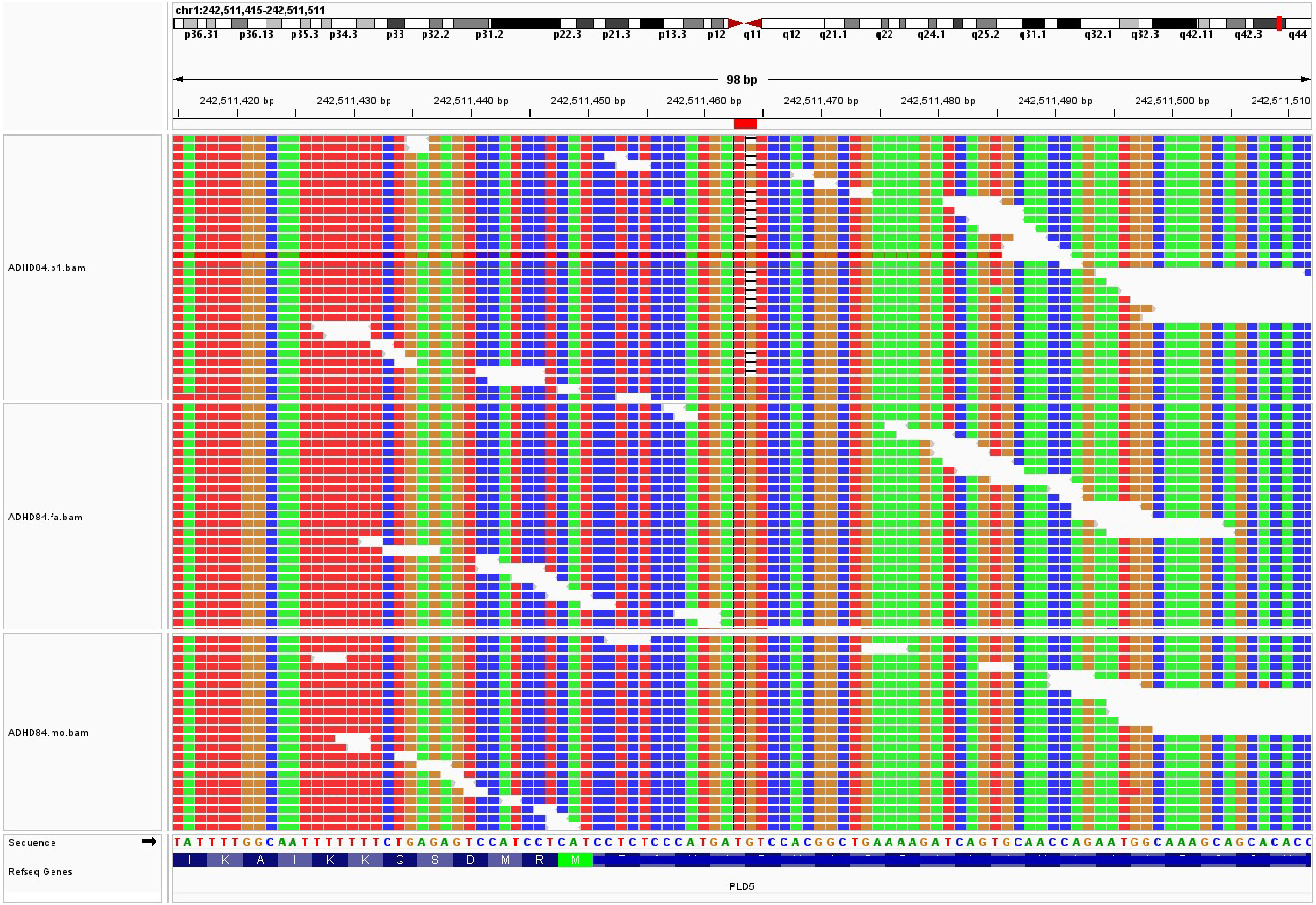

R. Chr16:24567213A>G (missense) for ADHD6.p1, ADHD6.fa, and ADHD6.mo.

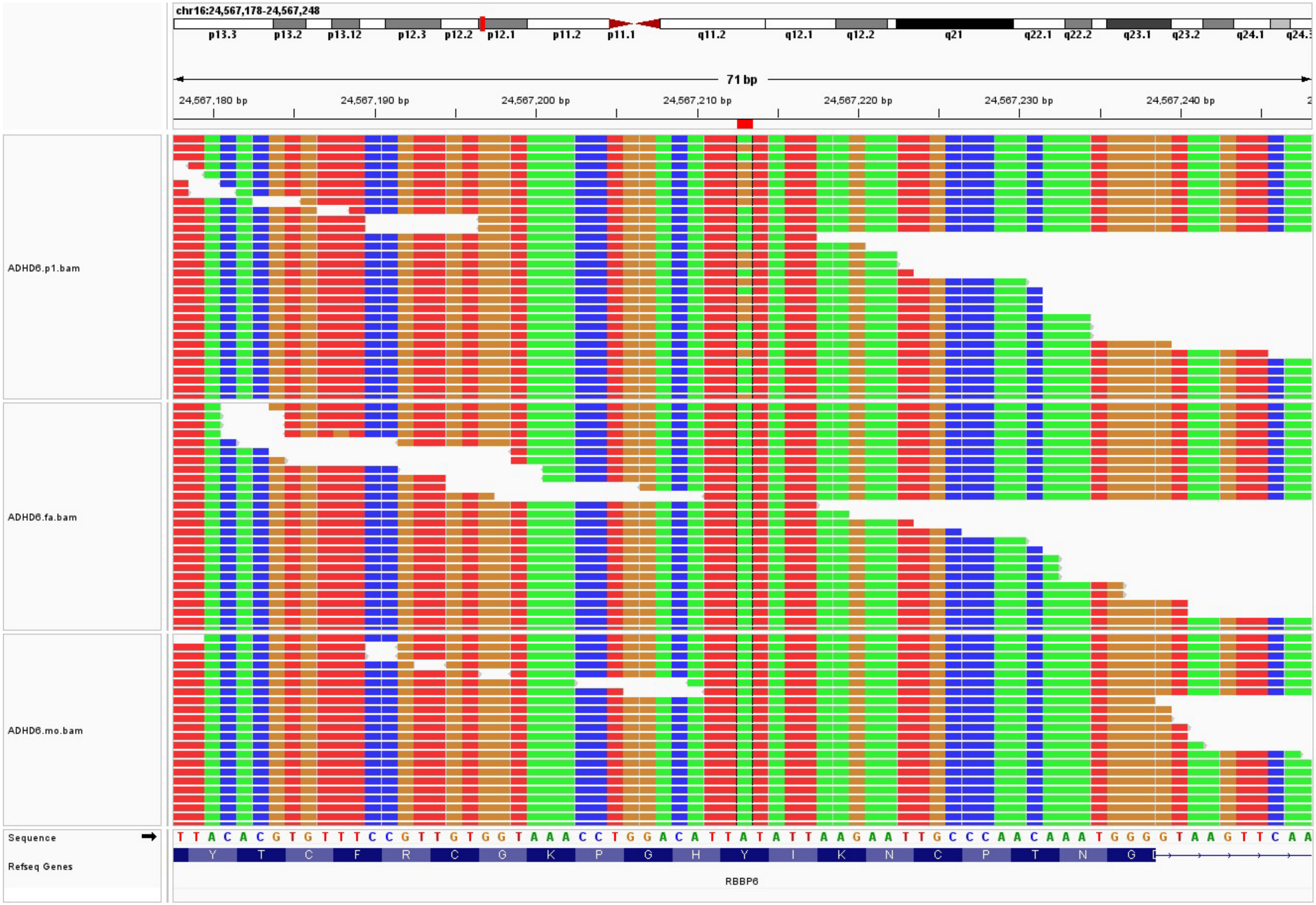

S. Chr15:49284809TG>T (frameshift deletion) for ADHD25.p1, ADHD25.fa, and ADHD25.mo.

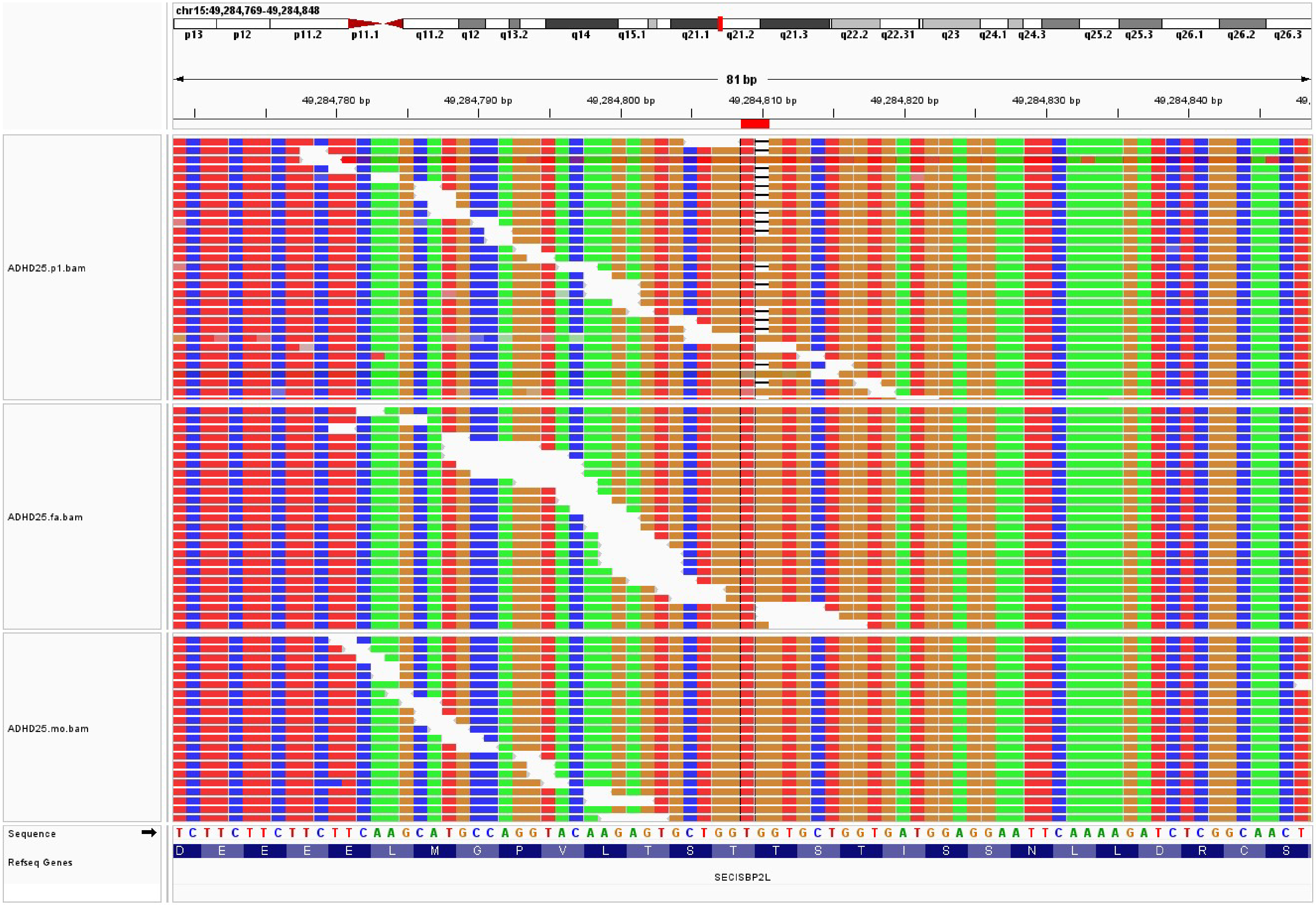

T. Chr3:136068009G>A (stopgain) for ADHD57.p1, ADHD57.fa, and ADHD57.mo.

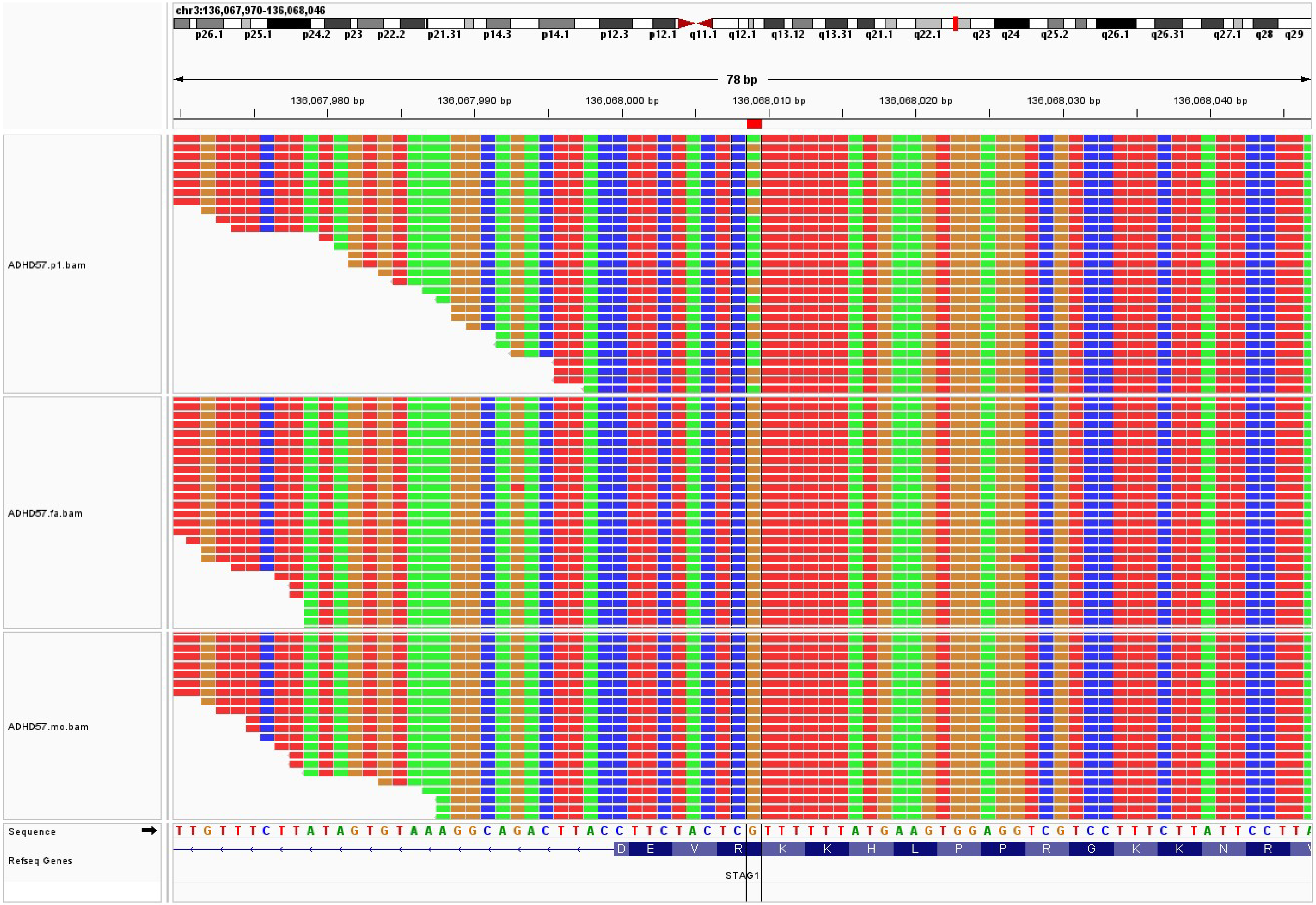

U. Chr7:138819468G>A (splicing) for ADHD71.p1, ADHD71.fa, and ADHD71.mo.

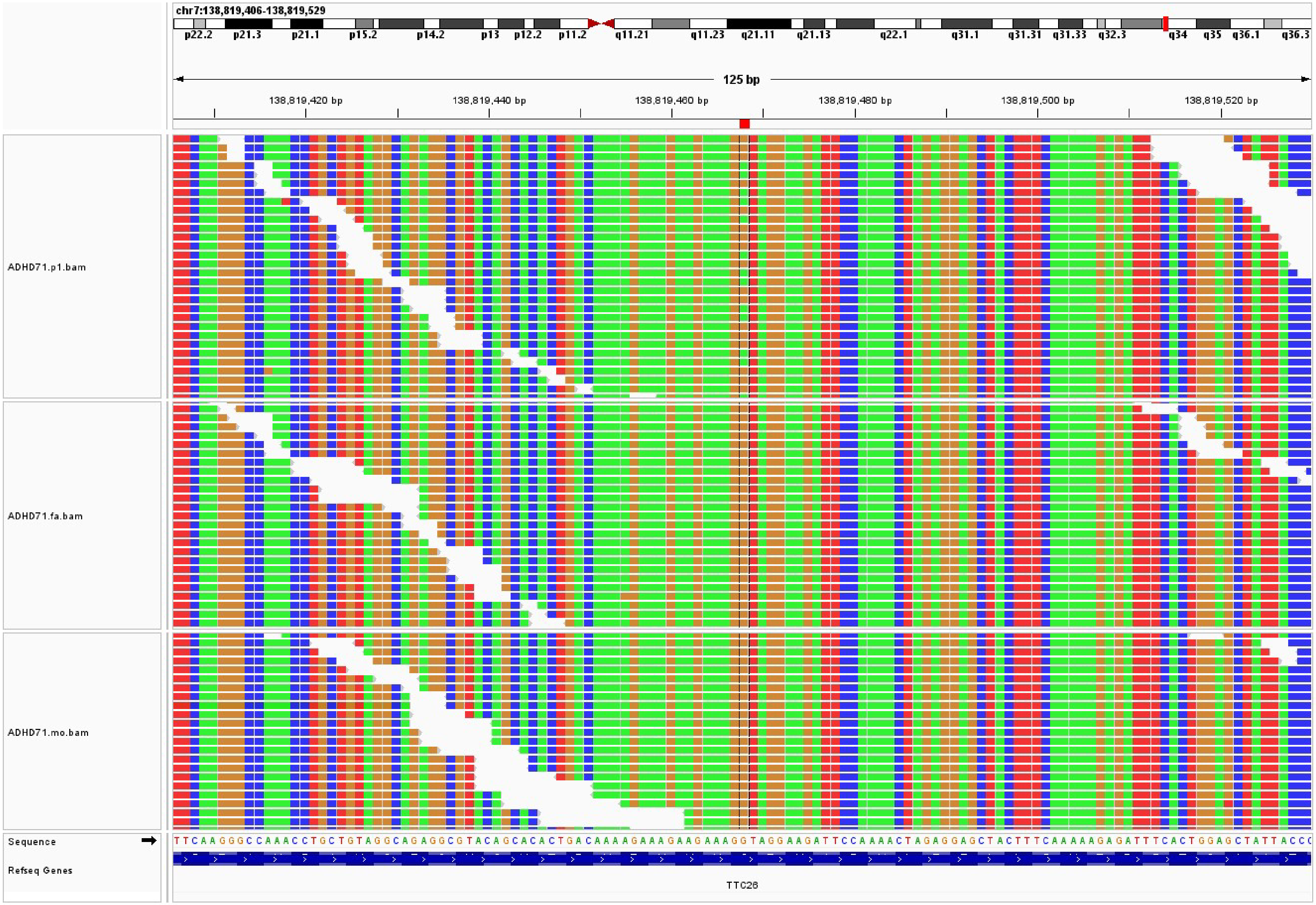

V. Chr6:30691669G>A (missense) for ADHD99.p1, ADHD99.fa, and ADHD99.mo.

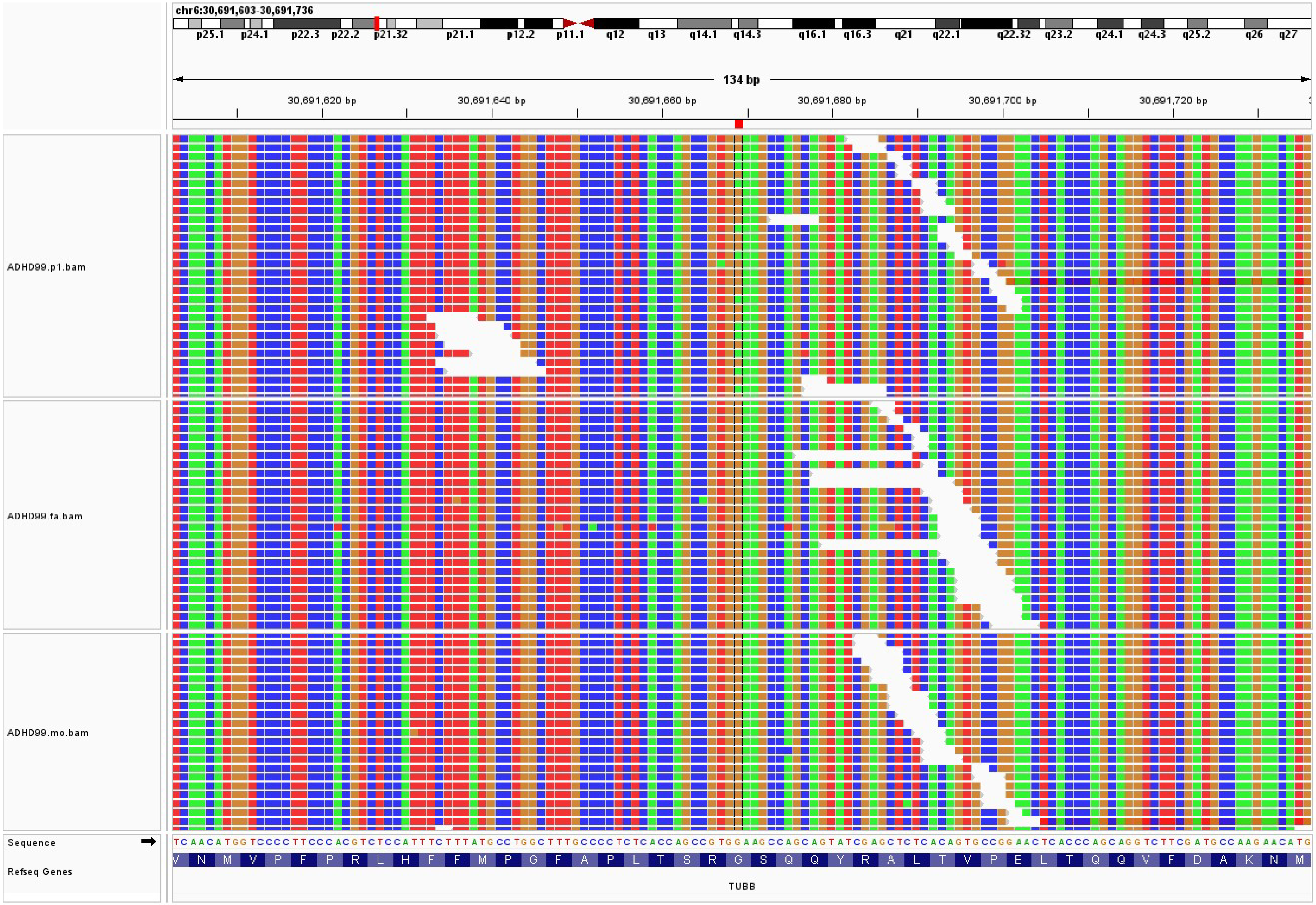

W. Chr10:75331247G>A (stopgain) for ADHD144.p1, ADHD144.fa, and ADHD144.mo.

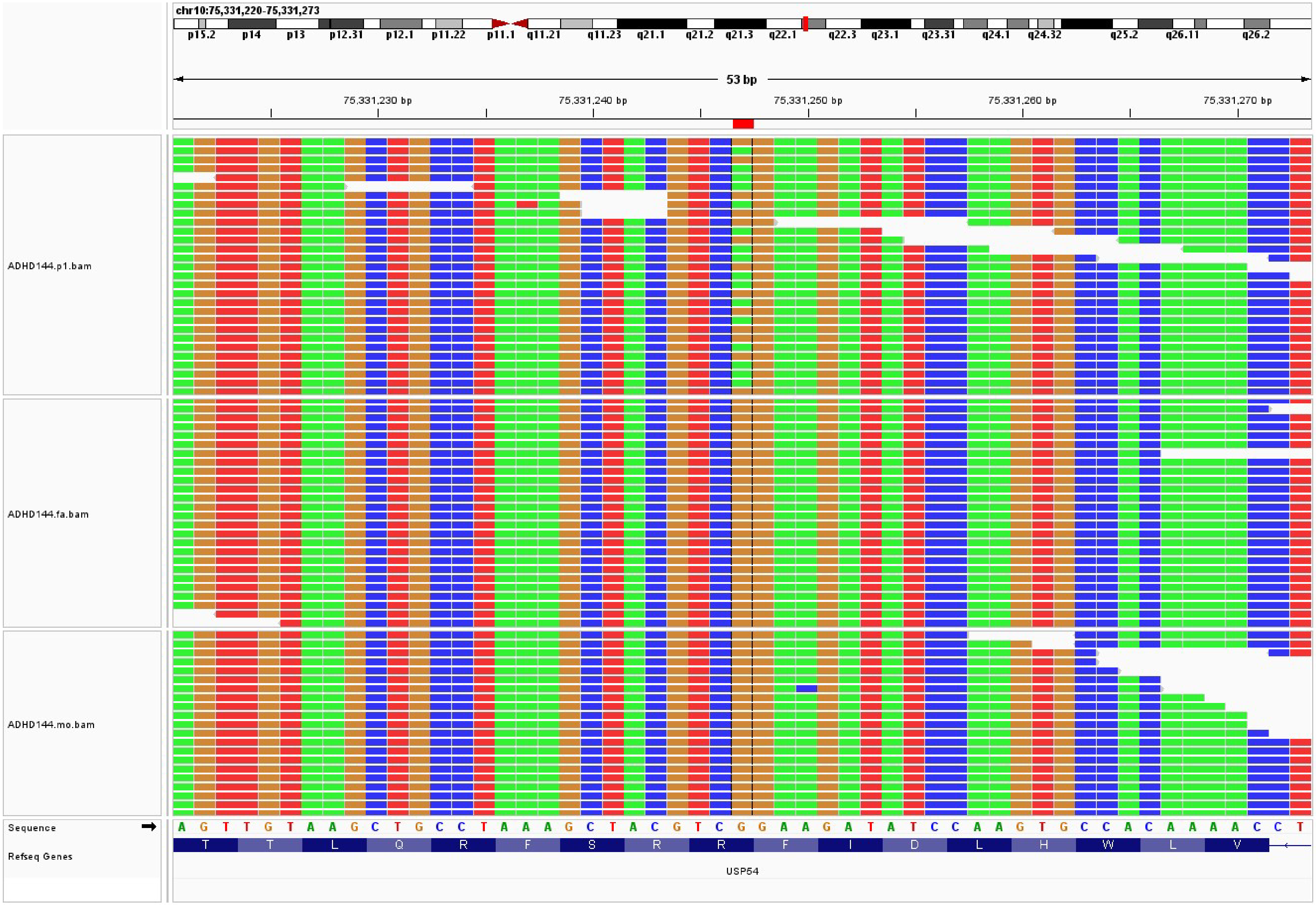

X. Chr14:75276497C>T (stopgain) for ADHD14.p1, ADHD14.fa, and ADHD14.mo.

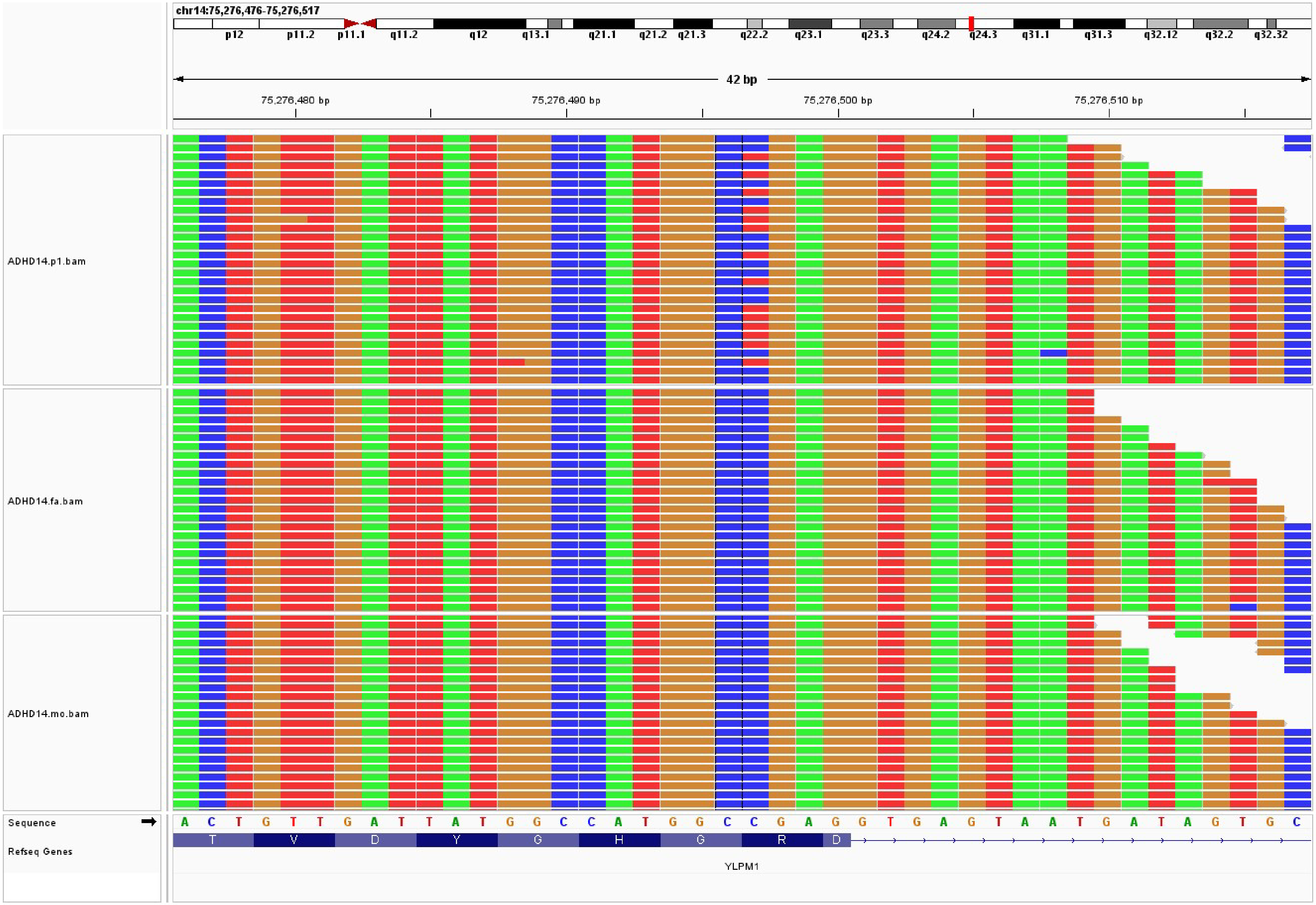

Supplementary Figure 2. Visualization of aligned sequencing reads for ultra-rare *de novo* variants identified in ADHD probands using Integrative Genomics Viewer (IGV, https://igv.org/[1,2]). For each variant, aligned reads from binary alignment map (.bam) files are shown for the proband, father, and mother. Figures are centered around the location of the variant (red bar), illustrating presence in the proband and absence in both parents. Variant details, including coordinates and annotation, are provided in Table 1 and Supplementary Data 2. Aligned sequencing reads are displayed for the following genomic coordinates, variants, and families:

A. Chr1:45251759CAGAG>C (frameshift deletion) for ADHD44.p1, ADHD44.fa, and ADHD44.mo.
B. Chr11:65623472T>C (missense) for ADHD69.p1, ADHD69.fa, and ADHD69.mo.
C. Chr10:125804342G>GGT (frameshift insertion) for ADHD95.p1, ADHD95.fa, ADHD95.mo.
D. Chr2:80808942C>T (missense) for ADHD37.p1, ADHD37.fa, and ADHD37.mo.
E. Chr5:11236867AC>A (frameshift deletion) for ADHD107.p1, ADHD107.fa, and ADHD107.mo.
F. Chr11:65359271CATGG>C (frameshift deletion) for ADHD98.p1, ADHD98.fa, and ADHD98.mo.
G. Chr2:55071273C>T (stopgain) for ADHD61.p1, ADHD61.fa, and ADHD61.mo.
H. Chr2:48035289G>A (missense) for ADHD86.p1, ADHD86.fa, and ADHD86.mo.
I. Chr5:180665146T>C (missense) for ADHD134.p1, ADHD134.fa, and ADHD134.mo.
J. Chr3:121435768A>C (stopgain) for ADHD117.p1, ADHD117.fa, and ADHD117.mo.
K. Chr1:202704703G>A (stopgain) for ADHD50.p1, ADHD50.fa, and ADHD50.mo.
L. Chr1:202711840TC>T (frameshift deletion) for ADHD58.p1, ADHD58.fa, and ADHD58.mo.
M. Chr1:62676185C>A (stopgain) for ADHD130.p1, ADHD130.fa, and ADHD.mo.
N. Chr11:78282471G>A (stopgain) for ADHD33.p1, ADHD33.fa, and ADHD33.mo.
O. Chr19:17337532G>T (stopgain) for ADHD71.p1, ADHD71.fa, and ADHD71.mo.
P. Chr11:77103522G>A (missense) for ADHD141.p1, ADHD141.fa, and ADHD141.mo.
Q. Chr1:242511463TG>T (frameshift deletion) for ADHD84.p1, ADHD84.fa, and ADHD84.mo.
R. Chr16:24567213A>G (missense) for ADHD6.p1, ADHD6.fa, and ADHD6.mo.
S. Chr15:49284809TG>T (frameshift deletion) for ADHD25.p1, ADHD25.fa, and ADHD25.mo.
T. Chr3:136068009G>A (stopgain) for ADHD57.p1, ADHD57.fa, and ADHD57.mo.
U. Chr7:138819468G>A (splicing) for ADHD71.p1, ADHD71.fa, and ADHD71.mo.
V. Chr6:30691669G>A (missense) for ADHD99.p1, ADHD99.fa, and ADHD99.mo.
W. Chr10:75331247G>A (stopgain) for ADHD144.p1, ADHD144.fa, and ADHD144.mo.
X. Chr14:75276497C>T (stopgain) for ADHD14.p1, ADHD14.fa, and ADHD14.mo.

Supplementary Figure 3. Visualization of aligned sequencing reads for putative rare and ultra-rare *de novo* damaging variants identified in ADHD parent-child trios. These two variants failed *in silico* confirmation and were not included in burden or downstream analyses.

A. Chr9:134398355G>GTCCAACAC (frameshift insertion) for ADHD8.p1, ADHD8.fa, and ADHD8.mo.

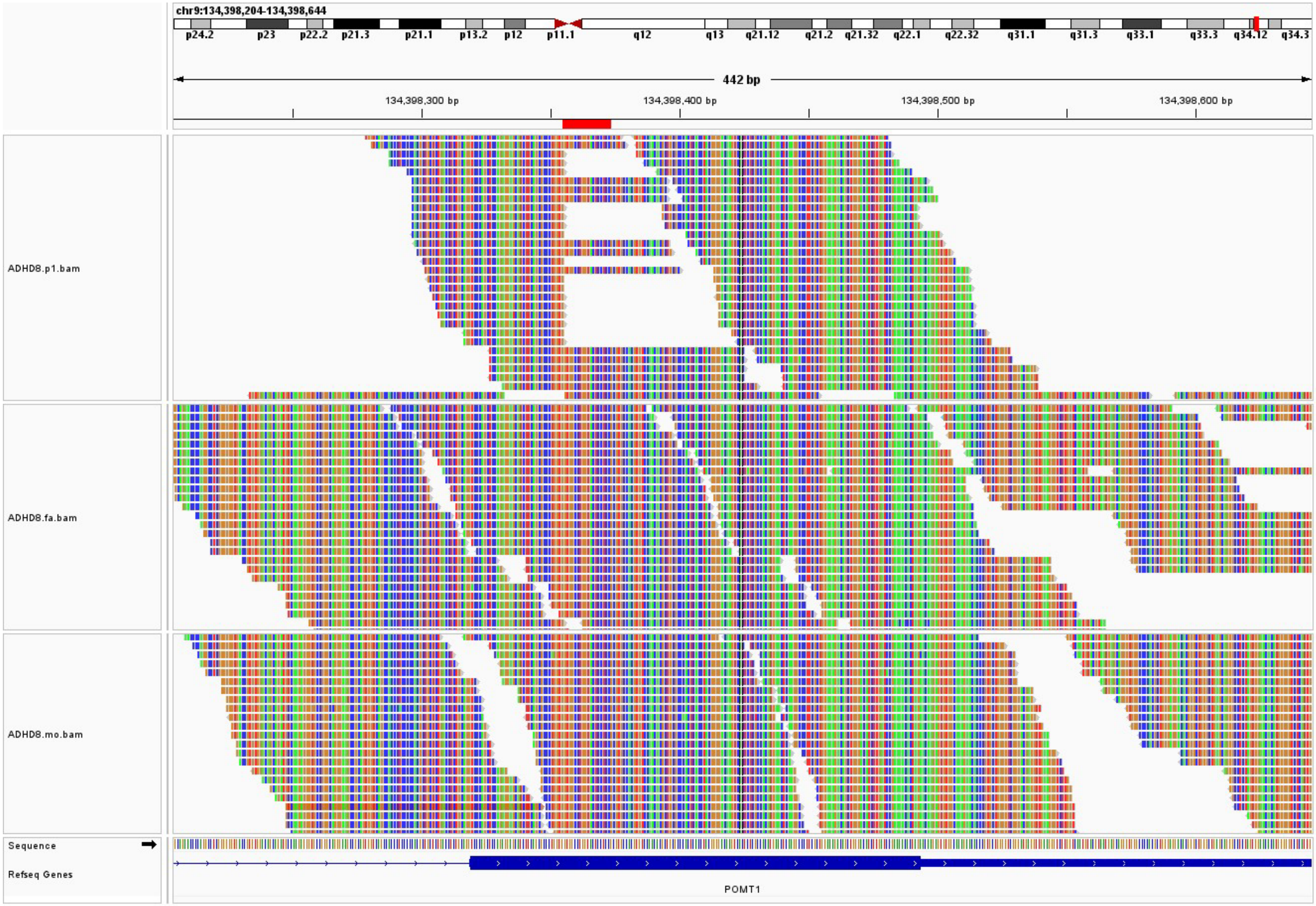

B. Chr8:10467603C>CA (frameshift insertion) for ADHD91.p1, ADHD91.fa, and ADHD91.mo.

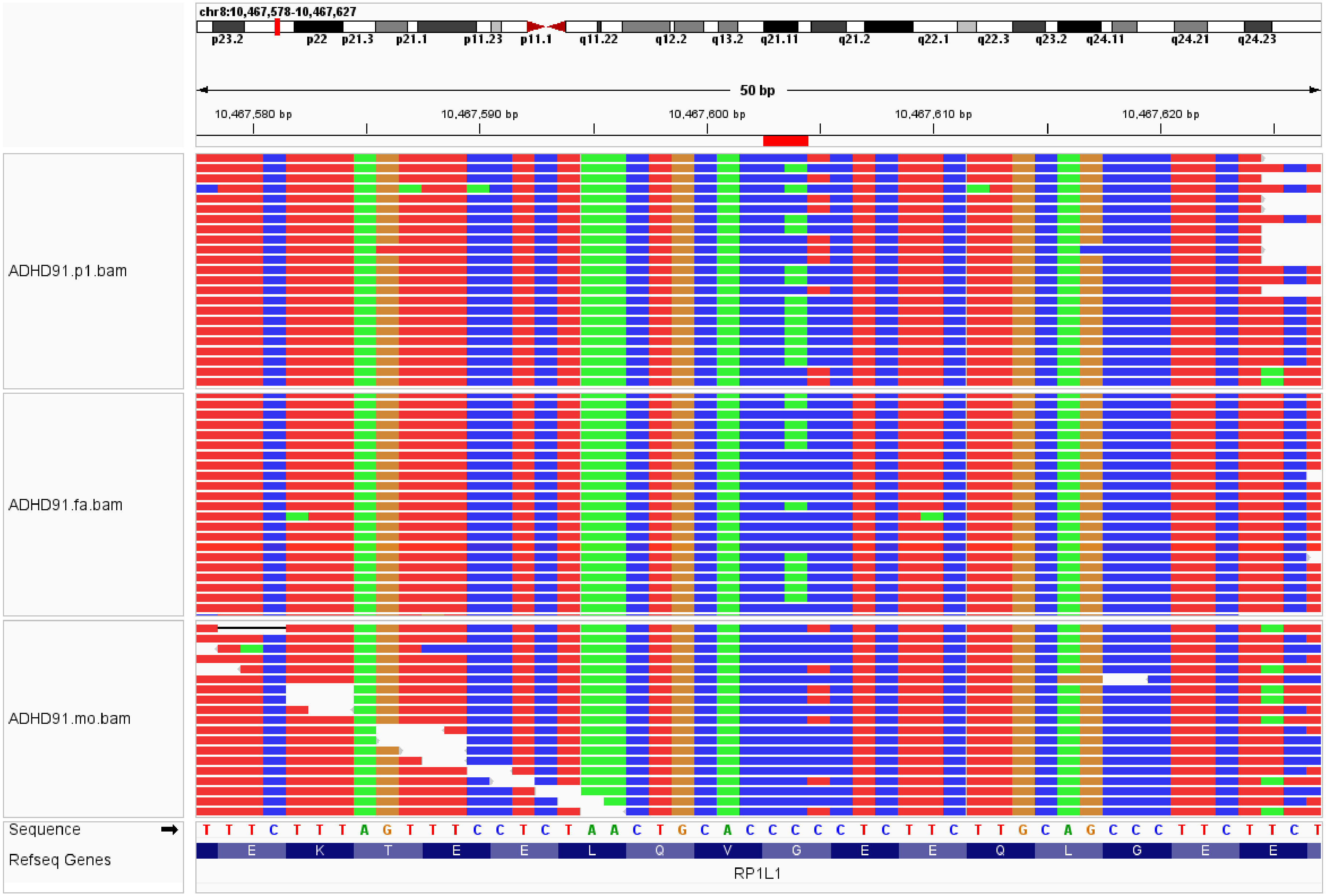

Supplementary Figure 3. Visualization of aligned sequencing reads for putative rare and ultra-rare *de novo* damaging variants called in 147 ADHD parent-child trios using Integrative Genomics Viewer (IGV, https://igv.org/[1,2]). For each variant, aligned reads from binary alignment map (.bam) files are shown for the proband, father, and mother. Figures are centered around the location of the putative variant (red bar). Two variants were called by GATK but did not confirm on visual inspection. Variant details, including coordinates and annotation, are provided in Supplementary Data 2. Aligned sequencing reads are displayed for the following genomic coordinates, variants, and families:

A. Chr9:134398355G>GTCCAACAC (frameshift insertion) for ADHD8.p1, ADHD8.fa, and ADHD8.mo.

B. Chr8:10467603C>CA (frameshift insertion) for ADHD91.p1, ADHD91.fa, and ADHD91.mo.

Supplementary Methods

We confirmed all rare *de novo* damaging variants entering into our analyses (Table 1, Supplementary Data 2) by visualizing aligned sequencing reads using the Integrative Genomics Viewer (IGV, https://igv.org/)[2] for the proband, father, and mother (Supplementary Figure 2). *In silico* visualization increases confidence in variant calls and reduces the risk of false positives[1]. Aggregate data from our earlier studies using the same analytical pipeline and the same stringent thresholds for calling *de novo* SNVs and indels support this approach[3,4]. In these studies, we confirmed 192/193 (99.5%) *de novo* SNVs and 7/8 (87.5%) *de novo* indels by Sanger sequencing, finding that *in silico* visualization was consistent with Sanger confirmation status. Although DNA from Simons Simplex Collection control subjects and from Genizon biobank ADHD samples were not available for confirmation by Sanger sequencing, prior Sanger validation of our *in silico* confirmation methods and our application of joint calling with stringent calling thresholds in cases and controls make it unlikely that our findings are biased by false positive calls.

Given the small sample size of female participants with ADHD, sex-based analyses were not performed *a priori*, so these data are not highlighted in our results. However, sex-stratified mutation rates were calculated in Supplementary Data 2.

Supplementary Discussion

Although no other gene beyond *KDM5B* was found to have more than one ultra-rare *de novo* gene-damaging mutation in unrelated individuals using our definition of PTV and Mis-D, in addition to the genes highlighted in the main Discussion, several additional genes harboring ultra-rare *de novo* damaging variants in ADHD are highlighted here. First, we identified a *de novo* PTV in *YPLM1*, which we identify as a potential risk gene for ADHD, based on FDR < 0.3 (**Table 1, Figure 2, Supplementary Data 3**). YLPM1 is involved in RNA binding and has been predicted to be involved in telomere maintenance, but to our knowledge, psychiatric manifestations related to *YLPM1* mutations have not been described previously. *CTNND2* (FDR=0.26) and *EML6* each had a *de novo* PTV variant in one individual with ADHD and a *de novo* missense variant in a second individual that was not predicted to be damaging according to MPC<2. However, it is interesting to note that these *de novo* missense variants were predicted to be possibly damaging (*EML6*) or probably damaging (*CTNND2*) using a different (less stringent) metric commonly used in WES studies, PolyPhen2-HDIV (**Supplementary Data 2**). Additionally, in the case-control dataset, another individual with ADHD was identified to have

a PTV in *CTNND2,* while no such variants were found in controls (**Supplementary Data 3**). *CTNND2* encodes an adhesive junction protein, and mutations have been previously associated with intellectual disability in Cri-du-Chat syndrome, ASD, and epilepsy[7-9]. Research suggests that *CTNND2* is important for the formation of dendritic spines and synapses[8].

Supplementary References

1 Robinson, J. T., Thorvaldsdóttir, H., Wenger, A. M., Zehir, A. & Mesirov, J. P. Variant Review with the Integrative Genomics Viewer. *Cancer Res* **77**, e31-e34 (2017). https://doi.org:10.1158/0008-5472.Can-17-0337

2 Robinson, J. T. *et al.* Integrative genomics viewer. *Nat Biotechnol* **29**, 24-26 (2011). https://doi.org:10.1038/nbt.1754

3 Cappi, C. *et al.* De novo damaging DNA coding mutations are associated with obsessive-compulsive disorder and overlap with Tourette's disorder and autism. *Biol Psychiatry* **87**, 1035-1044 (2020). https://doi.org:10.1016/j.biopsych.2019.09.029

4 Fernandez, T. V. *et al.* Primary complex motor stereotypies are associated with de novo damaging DNA coding mutations that identify KDM5B as a risk gene. *PLoS One* **18**, e0291978 (2023). https://doi.org:10.1371/journal.pone.0291978

5 Geis, T. *et al.* Clinical long-time course, novel mutations and genotype-phenotype correlation in a cohort of 27 families with POMT1-related disorders. *Orphanet Journal of Rare Diseases* **14**, 1-17 (2019).

6 Song, D. *et al.* Genetic variations and clinical spectrum of dystroglycanopathy in a large cohort of Chinese patients. *Clinical genetics* **99**, 384-395 (2021).

7 Medina, M., Marinescu, R. C., Overhauser, J. & Kosik, K. S. Hemizygosity of δ-catenin (CTNND2) is associated with severe mental retardation in cri-du-chat syndrome. *Genomics* **63**, 157-164 (2000).

8 Turner, T. N. *et al.* Loss of δ-catenin function in severe autism. *Nature* **520**, 51-56 (2015).

9 van Rootselaar, A.-F. *et al.* δ-Catenin (CTNND2) missense mutation in familial cortical myoclonic tremor and epilepsy. *Neurology* **89**, 2341-2350 (2017).
