# Supplementary material for: Rare de novo damaging DNA variants are enriched in attention-deficit/hyperactivity disorder and implicate risk genes
Source: Nat Commun. 2024 Jul 12;15:5870. doi: 10.1038/s41467-024-50247-7 (PMC11245598; doi:10.1038/s41467-024-50247-7)
Supplement: Supplementary file 3 — Description of Additional Supplementary Files [file 41467_2024_50247_MOESM3_ESM.pdf]

Description of Additional Supplementary Files

Supplementary Data 1: Metrics and quality control information from whole-exome DNA sequencing data of parent-child trios. This dataset includes sample information and quality control metrics from parent-child trios with ADHD (n=152) and unaffected controls (n=788) (1.1). Additional exome metrics for ADHD cases (1.2) and the data from the principal components analysis (1.3-1.4) are included. A key of column headings is included.

Supplementary Data 2: Details of all rare de novo variants identified in 147 ADHD probands (2.1) and 780 controls (2.2) passing quality control. Rare variants are defined by an allele frequency<0.001 in the non-neuro subset of the Genome Aggregation Database (gnomAD v2.2.1). Other tabs highlight gene-level overlap of rare de novo variants between cases and controls (2.3), exploratory sex-stratified de novo mutation rate comparisons (2.4), proportions of ultra-rare de novo variant types (2.5), and exploratory case-control de novo mutation rate comparisons using alternate missense pathogenicity and PTV constraint metrics (2.6). A key of column headings is included.

Supplementary Data 3: Gene-based test results combining the ultra-rare de novo damaging variants and independent case-control data. We used the Bayesian extension of the Transmission And De novo Association test (extTADA) to examine ultra-rare de novo protein-truncating variants (PTV) and missense variants predicted to be damaging (MPC score>2, Mis-D) from 147 ADHD parent-child trios, 3,206 ADHD cases, and 5,002 typically developing controls. We ran extTADA to calculate each gene's Bayes factor and q-values (false discovery rate, FDR). One gene, KDM5B, is classified as a high-confidence risk gene (FDR, false discovery rate < 0.1), and three genes, YLPM1, CTNND2, and GNB2L1, are classified as potential risk genes (FDR<0.3). A key of column headings is included.

Supplementary Data 4: Overlap between genes harboring ultra-rare de novo damaging variants in ADHD probands and genes identified in other DNA sequencing studies of parent-child trios, including a study by Fu et al., 2022 (4.1), and studies catalogued in the Gene4Denovo database. Gene4Denovo integrates de novo mutations from 68,404 individuals across 37 different phenotypes, including several neuropsychiatric conditions, but not including ADHD. We assessed the overlap between the Gene4Denovo candidate gene list (release version updated 07/08/2022) and our list of ultrarare de novo damaging variants, which included protein-truncating variants (PTVs) and missense variants predicted to be damaging (MPC score>2, Mis-D). A key of column headings is included.

Supplementary Data 5: Overlap between the genes harboring ultra-rare de novo damaging variants in ADHD probands and loci mapped to genes in genome-wide association studies (GWAS) of neuropsychiatric conditions from the GWAS Catalog. Ultra-rare de novo damaging variants include protein-truncating variants (PTVs) and missense variants predicted to be damaging (MPC score>2, Mis-D). The GWAS Catalog identifies studies through weekly PubMed searches and extracts data for single nucleotide polymorphisms (SNPs) with p < 1 x 10- 5 in the overall (initial GWAS + replication) population. A key of column headings is includeed.

Supplementary Data 6: Genes harboring ultrarare de novo damaging variants in ADHD cases are enriched for pathway and gene ontologybased sets. Ultra-rare de novo damaging variants include protein truncating variants (PTVs) and missense variants predicted to be damaging by (MPC score>2, Mis-D). We used ConsensusPathDB to conduct an exploratory analysis of pathways and gene ontology enrichment among 23 genes (see main Table 1). All sets with a p-value <0.01 are listed. A key of column headings is included.
